# Suppression of mutant Kirsten-RAS (KRAS^G12D^)-driven pancreatic carcinogenesis by dual-specificity MAP kinase phosphatases 5 and 6

**DOI:** 10.1038/s41388-022-02302-0

**Published:** 2022-04-13

**Authors:** Andrew M. Kidger, Mark K. Saville, Linda K. Rushworth, Jane Davidson, Julia Stellzig, Motoharu Ono, Ludwig A. Kuebelsbeck, Klaus-Peter Janssen, Bernhard Holzmann, Jennifer P. Morton, Owen J. Sansom, Christopher J. Caunt, Stephen M. Keyse

**Affiliations:** 1 Stress Response Laboratory, Jacqui Wood Cancer Centre, Division of Cellular and Systems Medicine, School of Medicine, University of Dundee, Dundee, DD1 9SY UK; 2 Department of Surgery, School of Medicine, Klinikum Rechts der Isar, Technical University of Munich, Munich, Germany; 3 Institute of Cancer Sciences, Garscube Estate, Switchback Road, Glasgow, G61 1QH UK; 4 CRUK Beatson Institute, Garscube Estate, Switchback Road, Glasgow, G61 1BD UK; 5 Department of Biology and Biochemistry, University of Bath, Claverton Down, Bath, BA2 7AY UK

**Keywords:** Oncogenes, Predictive markers

## Abstract

The cytoplasmic phosphatase DUSP6 and its nuclear counterpart DUSP5 are negative regulators of RAS/ERK signalling. Here we use deletion of either *Dusp5* or *Dusp6* to explore the roles of these phosphatases in a murine model of KRAS^G12D^-driven pancreatic cancer. By 56-days, loss of either DUSP5 or DUSP6 causes a significant increase in KRAS^G12D^-driven pancreatic hyperplasia. This is accompanied by increased pancreatic acinar to ductal metaplasia (ADM) and the development of pre-neoplastic pancreatic intraepithelial neoplasia (PanINs). In contrast, by 100-days, pancreatic hyperplasia is reversed with significant atrophy of pancreatic tissue and weight loss observed in animals lacking either DUSP5 or DUSP6. On further ageing, *Dusp*6^−/−^ mice display accelerated development of metastatic pancreatic ductal adenocarcinoma (PDAC), while in *Dusp5*^−/−^ animals, although PDAC development is increased this process is attenuated by atrophy of pancreatic acinar tissue and severe weight loss in some animals before cancer could progress. Our data suggest that despite a common target in the ERK MAP kinase, DUSP5 and DUSP6 play partially non-redundant roles in suppressing oncogenic KRAS^G12D^ signalling, thus retarding both tumour initiation and progression. Our data suggest that loss of either DUSP5 or DUSP6, as observed in certain human tumours, including the pancreas, could promote carcinogenesis.

## Introduction

Dual-specificity phosphatase 5 (DUSP5), a nuclear MAP kinase phosphatase (MKP) and dual-specificity phosphatase 6 (DUSP6, also known as MAP Kinase Phosphatase-3 or MKP-3), a cytoplasmic MKP, are both extracellular signal-regulated kinase (ERK)-specific phosphatases, which are transcriptionally induced in response to RAS/ERK signalling in mammalian cells and tissues and thus act as classical negative feedback regulators of ERK activity [1–5]. In addition to their catalytic activity towards ERK1/2, both phosphatases bind tightly to ERK and can retain the inactive kinase in either the nucleus (DUSP5) or the cytoplasm (DUSP6), indicating that they act to regulate the spatiotemporal activity of this key growth factor regulated signalling pathway [3, 6, 7]. Because abnormal activation of RAS/ERK signalling is frequently observed in human cancers [8] and both *DUSP5* and *DUSP6* are often up-regulated in tumours and cancer cell lines, which harbour activating mutations in the RAS/ERK pathway [9–13], these enzymes have been presumed to negatively regulate the oncogenic potential of signalling and to be potential tumour suppressors.

Recent studies using knockout mice and cells derived from them have confirmed a key role for DUSP5 in the regulation of nuclear ERK activity and the suppression of mutant HRAS^Q61L^-driven DMBA/TPA-induced skin papillomas, confirming a tumour suppressor function for this phosphatase [14]. However, there is relatively little evidence linking DUSP5 with human cancers. DUSP5 expression has been reported to be down-regulated in gastric, colorectal and prostate cancers [15–17], where its loss is associated with a poorer prognosis. However, recent work using transgenic mice expressing DUSP5 in the intestine indicates that it is not a major regulator of intestinal homoeostasis, nor does its overexpression seem to counteract adenoma formation in the *Apc*^*Min/+*^ model of intestinal tumourigenesis, both processes that require active ERK signalling [18].

Loss of DUSP6 expression has been linked to disease progression in both mutant KRAS-driven pancreatic and non-small cell lung cancer. In the human pancreas, DUSP6 expression initially increases in early stage lesions, but is then epigenetically silenced with the lowest levels of DUSP6 found in poorly differentiated and invasive tumours [19–21]. In lung tumours, loss of DUSP6 is associated with increased clinical severity and histological grade [22]. More recently, deletion of *Dusp6* has been found to promote intestinal proliferation and to increase tumour burden in *Apc*^Min/+^ mice, again suggesting a tumour suppressive function [23]. However, evidence has also emerged that DUSP6 may be a positive regulator of carcinogenesis. DUSP6 is overexpressed in human glioblastoma, where it appears to cause cellular changes associated with invasion and metastasis and tumours derived from glioblastoma cells expressing DUSP6 grow significantly faster than non-expressing controls in mouse xenograft experiments [24]. DUSP6 has also been reported to facilitate the survival and transformation of pre-B cells by the *BCR-ABL1* Philadelphia chromosome rearrangement and mutant NRAS^G12D^, both of which drive acute lymphoblastic leukaemia (ALL) and to be essential for oncogenic transformation in mouse models of ALL [25]. Finally, DUSP6 has been identified as a potential synthetic lethal target in melanoma cell lines, which harbour mutant *BRAF*^*V600E*^ and express high levels of this phosphatase [26].

We have used mice harbouring a conditional *Kras*^*G12D*^ knock-in allele silenced by a floxed STOP transcriptional cassette (*LSL*-*Kras*^*G12D*^) in combination with pancreas-specific expression of Cre recombinase under the control of the *Ptf1a/P48* promoter. Approximately 90% of pancreatic cancers harbour activating mutations in *KRAS* and this murine model faithfully recapitulates the full spectrum of histological lesions that characterise the progression of human pancreatic carcinogenesis, giving rise to pancreatic ductal adenocarcinomas (PDAC) that display desmoplastic stroma and inflammatory responses closely resembling those observed in human patients [27]. This model has been widely used to study loss or mutation of tumour suppressor loci found in human pancreatic cancers, with such compound strains resulting in accelerated pancreatic tumour progression and in the induction of invasive and metastatic cancer [28, 29]. Here we combine it with conditional alleles of either *Dusp5* or *Dusp6* to explore the roles of these MKP’s in pancreatic carcinogenesis.

## Results

### Mutant KRAS^G12D^ leads to up-regulation of both *Dusp5* and *Dusp6* in MEFs and murine pancreas

**Fig. 1:**
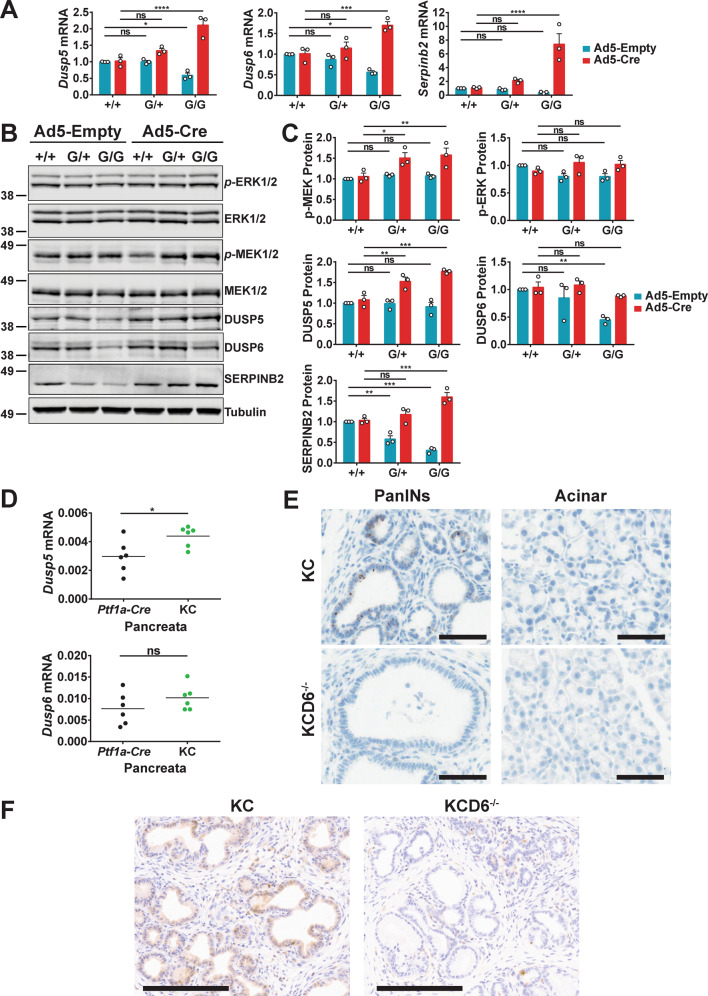
Mutant KRAS^G12D^ leads to up-regulation of both DUSP5 and DUSP6 in MEFs and murine pancreas. Three sets of *Kras*^*+/+*^(+/+), *Kras*^*LSL-G12D/+*^ (G/+) and *Kras*^*LSL-G12D/LSL-G12D*^ (G/G) MEF lines derived from independent littermates were infected with either empty adenovirus (Ad5-Empty) or adenoviral-*Cre* (Ad5-Cre) for 48 h prior to either RNA isolation (**A**), or immunoblotting using the indicated antibodies (**B**, **C**). **A** TaqMan RT-qPCR analysis of the indicated transcripts showing the fold change in mRNA levels relative to wild type cells infected with empty adenovirus. **B** A Western blot representative of three independent experiments is shown, alongside graphs (**C**) showing the fold change in protein levels relative to wild type cells infected with empty adenovirus. The tubulin blot is shown as a representative loading control (*p*-ERK1/2/ERK1/2). For all quantitative data mean values from three independent experiments (*n* = 3) ± SEM are shown, *p < 0.05, **p < 0.01, ***p < 0.001, ****p < 0.0001 using two-way ANOVA and Bonferroni post hoc test, comparing *Kras* genotypes. **D** TaqMan qRT-PCR analysis showing mRNA levels of *Dusp5* or *Dusp6*, relative to Beta-actin (*Actb*), following RNA isolation from 100-day pancreata of the indicated cohorts. Mean values are shown from 6 pancreata per genotype (*n* = 6), ns not significant, *p < 0.05, using an unpaired t-test. **E** Representative images of RNAscope in situ hybridisation for *Dusp6* transcripts in 100-day pancreatic sections from the indicated cohorts. (Scale bars, 60 µM.) The expression of *Dusp6* transcripts is associated with KRAS^G12D^-induced Pancreatic Intraepithelial Neoplasia (PanINs, upper left panel), while acinar tissue (upper right panel) displays low or undetectable levels of *Dusp6* transcripts. The specificity of the RNAscope probe is demonstrated by the absence of staining in KCD6^−/−^ tissue (lower panels). **F** Representative images of DUSP6 immunohistochemistry in 100-day pancreatic sections showing cytoplasmic DUSP6 staining in pre-neoplastic PanINs from KC (left panel), but not KCD6^−/−^ (right panel) mice. (Scale bars, 200 µM.) Cohorts consisted of the following genotypes: *Kras*^*LSL-G12D/+*^*; Ptf1a-Cre; Dusp*^*+/+*^ (KC), *Kras*^*LSL-G12D/+*^*; Ptf1a-Cre; Dusp6*^*fl/fl*^ (KCD6^−/−^).

We previously showed that ectopic expression of mutant HRAS^Q61L^ in MEFs induced the expression of DUSP5 [30]. To explore the relationship between endogenous expression of mutant KRAS^G12D^, a major driver of pancreatic cancer development, and the expression of DUSP5 and DUSP6 we used adenoviral-*Cre*-mediated recombination in MEFs derived from littermate wild type *Kras*^*+/+*^ (+/+), heterozygous *LSL-Kras*^*G12D/+*^ (G/+) and homozygous *LSL-Kras*^*G12D/G12D*^ (G/G) embryos. As expected, the replacement of both copies of the WT *Kras* allele with *LSL-Kras*^*G12D/G12D*^, which is a null allele, causes a decrease in expression at the mRNA level for the ERK-responsive genes *Dusp5*, *Dusp6* and *SerpinB2* in cells treated with empty adenovirus. However, *Cre*-mediated recombination and expression of KRAS^G12D^ results in increased mRNA expression of all three genes and for homozygous (G/G) MEFs this reaches levels significantly above those seen in WT cells (Fig. 1A). This is reflected in increased levels of protein expression for both DUSP5 and SERPINB2, while DUSP6 seems less affected (Fig. 1B, C). The latter changes are accompanied by significantly increased levels of activated MEK (*p*-MEK), but no corresponding changes in ERK phosphorylation (*p*-ERK) (Fig. 1B, C), indicating that negative feedback mechanisms are acting upon ERK1/2 itself to prevent increased activation. The latter finding is in agreement with previous studies in which endogenous expression of KRAS^G12*D*^ was shown to have little effect on *p*-ERK levels [31]. Consistent with previous studies demonstrating that both phosphatases are classical negative feedback regulators of the RAS/ERK pathway [2, 4], KRAS^G12*D*^-induced *Dusp5* and *Dusp6* expression was greatly reduced by inhibition of MEK, but not PI3-kinase activity in *LSL-Kras*^*G12D/+*^ MEFs (Fig. S1A–C).

Next, we isolated pancreata from 100-day-old *Kras*^*+/+*^; *Ptf1a-Cre; Dusp*^*+/+*^ (Ptf1a-Cre) and *LSL-Kras*^*G12D/+*^*; Ptf1a-Cre; Dusp*^*+/+*^ (KC) mice and using RT-qPCR analysis determined that both *Dusp5* and *Dusp6* mRNA levels were increased in response to expression of KRAS^G12D^ (Fig. 1D). As mRNA was harvested from the whole organ, in which only a proportion of tissue will be undergoing KRAS^G12D^-induced transformation, these assays probably underestimate the expression of these phosphatases in KRAS^G12D^-expressing tissue. The latter is confirmed, at least for DUSP6, by RNAscope and IHC staining in pancreata from 100-day-old *LSL-Kras*^*G12D/+*^ *Ptf1a-Cre; Dusp*^*+/+*^, (KC) and, *LSL-Kras*^*G12D/+*^ *Ptf1a-Cre; Dusp6*^*fl/fl*^ (KCD6^−/−^) mice. *Dusp6* transcripts are only found associated with Pancreatic Intraepithelial Neoplasia (PanINs), with no expression detected in acinar tissue (Fig. 1E). This correlates exactly with cytoplasmic DUSP6 protein expression detected by IHC in these pre-neoplastic pancreatic lesions (Fig. 1F). Our results in murine pancreas agree with previous studies in which expression of DUSP6 was detected in these early lesions in human patients [21], and confirm that expression of this phosphatase is indeed associated with the early stages of KRAS^G12D^-induced pancreatic cancer development.

### Loss of either DUSP5 or DUSP6 promotes increased KRAS^G12D^-driven initiation of pancreatic carcinogenesis

Mice lacking either DUSP5 [14] or DUSP6 [32] have previously been shown to be viable and fertile. To determine if loss of either phosphatase had any consequences for normal pancreatic development, we compared pancreata isolated from wild type, *Dusp5*^−/−^, *Dusp6*^−/−^ and KC mice at 5 months of age. Organs from mice lacking either *Dusp5* or *Dusp6* exhibited normal size/weight when compared to WT, while KC mice expressing mutant KRAS^G12D^ in the pancreas showed the expected hyperplasia and increase in organ weight as reported previously [27]. Furthermore, the normal disposition of tissue types and structures, including islets of Langerhans (endocrine tissue), acinar cells (exocrine tissue) and pancreatic ducts was seen in pancreata lacking either phosphatase (Fig. S2A, B) and no abnormal ducts or pre-neoplastic lesions were detected. Taken together, these results indicate that loss of either DUSP5 or DUSP6 alone does not affect either pancreatic development or predispose to pancreatic carcinogenesis.

**Fig. 2:**
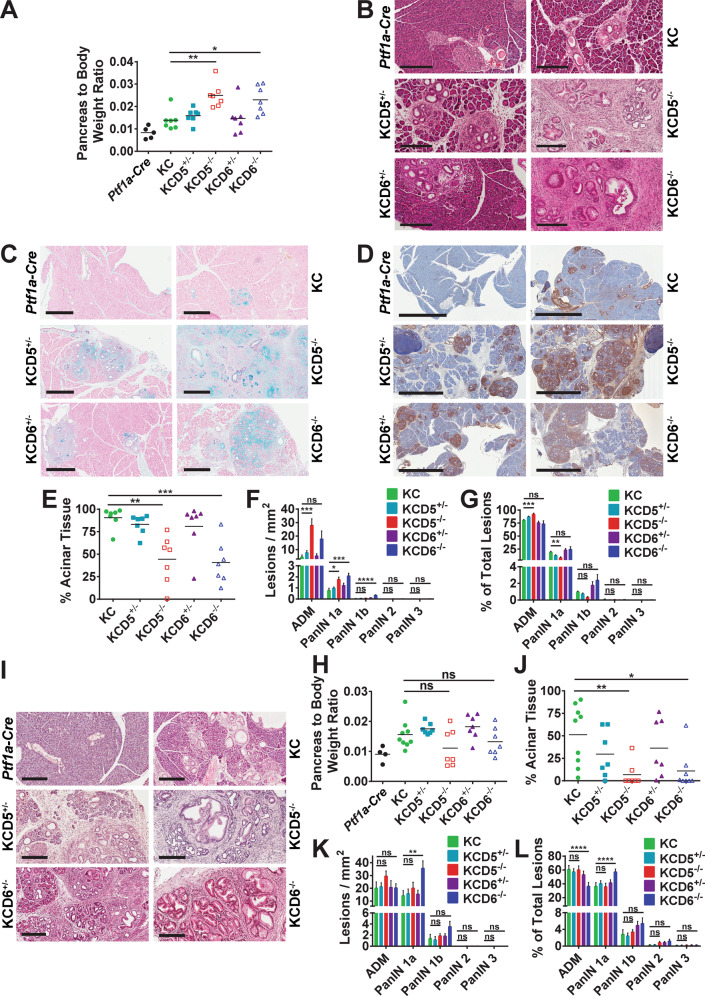
Loss of either DUSP5 or DUSP6 promotes increased KRAS^G12D^-driven initiation of pancreatic carcinogenesis. Pancreata from 56 (**A**) or 100-day (**H**) age-matched mice of the indicated cohorts were harvested and their pancreas to body weight ratios calculated. Cohorts consisted of the following genotypes: *Kras*^*+/+*^; *Ptf1a-Cre*; *Dusp*^*+/+*^ (Ptf1a-Cre), *Kras*^*LSL-G12D/+*^*; Ptf1a-Cre; Dusp*^*+/+*^ (KC), *Kras*^*LSL-G12D/+*^*; Ptf1a-Cre; Dusp5*^*+/fl*^ (KCD5^+/−^), *Kras*^*LSL-G12D/+*^*; Ptf1a-Cre; Dusp5*^*fl/fl*^ (KCD5^−/−^), *Kras*^*LSL-G12D/+*^*; Ptf1a-Cre; Dusp6*^*+/fl*^ (KCD6^+/−^) and *Kras*^*LSL-G12D/+*^*; Ptf1a-Cre; Dusp6*^*fl/fl*^ (KCD6^−/−^). Individual data points and mean values are shown, *n* = 7–9 (Ptf1a-Cre controls *n* = 4–5), ns not significant, *p < 0.05, **p < 0.01, using one-way ANOVA and Bonferroni post hoc test. Representative images of H&E (**B**), Alcian Blue/Nuclear Fast Red (**C**) and α-smooth muscle actin (αSMA) IHC (**D**) stained pancreata from 56-day age-matched mice of the indicated cohorts. (Scale bars, 200 μm, 500 μm and 2 mm, respectively.) Quantification of the pancreatic precursor lesion development in 56 (**E**–**G**) or 100-day (**J**–**L**) age-matched pancreata of the indicated cohorts. **E**, **J** Percentage of acinar tissue remaining in the pancreata of each cohort following KRAS^G12D^-driven ADM and PanIN initiation. **F**, **K** Total number of ADMs and PanINs of all histological grades per mm^2^ in the indicated cohorts. **G**, **L** Quantification of the number of pancreatic cancer precursor lesions, divided into each histological grade, expressed as a percentage of the total number of lesions per cohort. Quantification was performed on one representative section per mouse, following serial sectioning of the pancreas. Mean values ± SEM are shown, *n* = 7–9, ns not significant, *p < 0.05, **p < 0.01, ***p < 0.001, ****p < 0.0001 using one-way ANOVA and Bonferroni post hoc test. **I** Representative images of H&E stained pancreata from 100-day age-matched mice of the indicated cohorts. (Scale bars, 200 μm).

To study the effects of deleting either *Dusp5* or *Dusp6* on pancreatic carcinogenesis, we generated aged-matched cohorts of KC mice, which were wild type, heterozygous or homozygous for floxed alleles of either *Dusp5* (KCD5^+/−^;KCD5^−/−^) or *Dusp6* (KCD6^+/−^; KCD6^−/−^), along with Ptf1a-Cre controls. Animals were then sacrificed at 56-days and the pancreata were isolated, weighed, sectioned and stained to reveal any morphological changes. At 56-days, both KCD5^−/−^ and KCD6^−/−^ mice showed an increased pancreas to body weight ratio compared to KC animals (Fig. 2A). KRAS^G12D^-induced precursor lesions arise primarily through a process of acinar dedifferentiation and acinar to ductal metaplasia (ADM) with progression of ADM to PanINs [29, 33, 34]. ADM presents as proliferative tubular structures consisting of swollen acinar cells with a ductal appearance surrounded by reactive stroma. PanINs are classified PanIN1-3 according to the degree to which these ductal lesions exhibit progressive cytological and architectural atypia. Histological analysis revealed an increased burden of ADM and PanINs in mice lacking either DUSP5 or DUSP6 in the presence of KRAS^G12D^ when compared with KRAS^G12D^ expression alone (Figs. 2B and S3A). The increased burden of PanINs is clearly seen in alcian blue stained sections, which reveal the characteristic high levels of mucin secretion [27] and also by the presence of a dense reactive stroma which forms around these lesions and can be visualised using IHC detection of α-smooth muscle actin (Fig. 2C, D).

Quantification of these morphological changes reveals that there is a significant decrease in the levels of normal acinar tissue in both KCD5^−/−^ and KCD6^−/−^ mice compared to KC (Fig. 2E). This is accompanied by a marked increase in ADM in both KCD5^−/−^ and KCD6^−/−^ pancreata (Fig. 2F). Although at this stage, the vast majority of the pre-neoplastic lesions in all cohorts are classified as ADM, quantification by histological grade reveals that both KCD5^−/−^ and KCD6^−/−^ pancreata display an increase in the numbers of PanINs per mm^2^ when compared to KC mice, with KCD5^−/−^ mice showing a significant increase in both ADM and PanIN1a while KCD6^−/−^ animals show a significant increase in both PanIN1a and PanIN1b (Fig. 2F). When histological grade is quantified as a proportion of total lesions the only significant changes seen are an increase in ADM and decrease in PanIN1a on loss of DUSP5 (Fig. 2G).

To further investigate the effects of DUSP5 and DUSP6 loss on the progression of KRAS^G12D^-induced PanINs, we generated a second age-matched cohort that was sacrificed at 100-days. Interestingly, pancreas to body weight ratios amongst the KCD5^−/−^ and KCD6^−/−^ mice, which had significantly increased relative to KC mice at 56-days, had now declined somewhat (Fig. 2H). Histological analysis and quantification showed an even greater decrease in the proportion of healthy acinar tissue relative to KC mice, with the KCD5^−/−^ and KCD6^−/−^ organs displaying almost complete loss of normal acinar tissue and its replacement by ADM, PanINs and reactive stroma (Figs. 2I, J and S3B). This wholesale loss of acinar tissue most probably underpins the reduction in relative organ weight seen in the KCD5^−/−^ and KCD6^−/−^ mice at this time-point. Interestingly, qualitative differences began to emerge between the KCD5^−/−^ and KCD6^−/−^ animals in this cohort with H&E stained sections revealing that while KCD6^−/−^ pancreata display an increased number of tightly packed PanINs covering a larger area of the tissue, KCD5^−/−^ organs, despite a similar loss of acinar tissue, display more diffuse PanIN development with a significantly larger area of ADM and reactive stroma (Figs. 2I and S3B). Quantitative analysis reinforces this observation, revealing that KCD6^−/−^ mice exhibit a significantly higher number of PanIN1a per mm^2^ (Fig. 2K) and a significantly higher proportion of PanIN1a compared with either KC or KCD5^−/−^ mice. While not significant, a higher proportion of PanIN 1b and PanIN2 are also found in KCD6^−/−^ mice, although numbers are relatively low at this time point (Fig. 2L). The conclusion from this staged analysis is that while loss of either DUSP5 or DUSP6 promotes KRAS^G12D^-driven ADM and PanIN development, the loss of DUSP6 seems more able to promote accelerated progression of these lesions to higher histological grades. This could be due to differential effects of DUSP loss on the balance between tumour development and the progression (or resolution) of pancreatic atrophy.

**Fig. 3:**
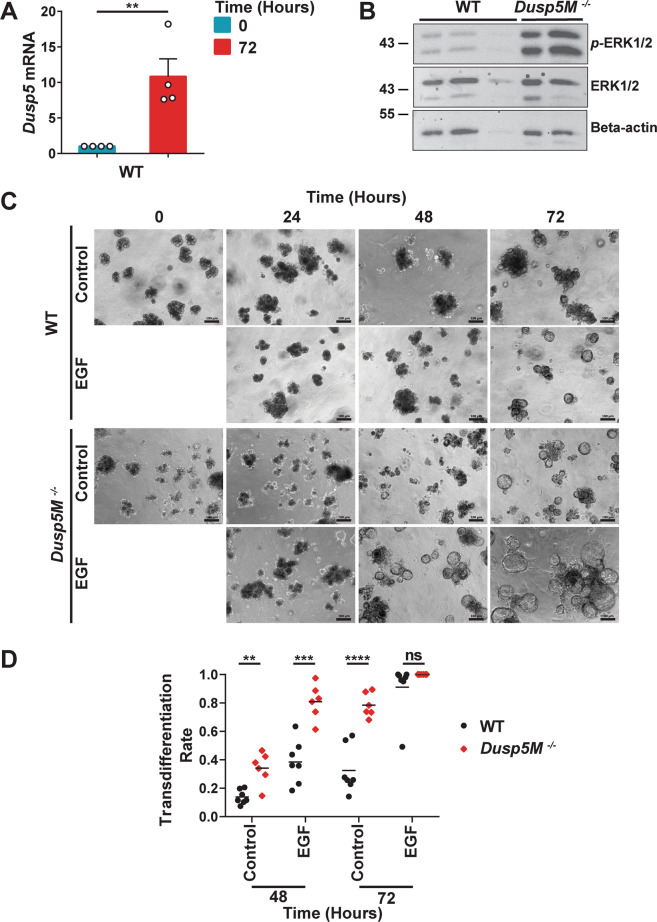
Loss of DUSP5 results in increased levels of activated ERK1/2 and accelerates the transdifferentiation of pancreatic acinar cells in vitro. **A** TaqMan RT-qPCR analysis showing mRNA levels of *Dusp5*, relative to Beta-actin (*Actb*) following RNA isolation from 3D acinar cultures at 0 and 72 h. Mean values from four independent experiments (*n* = 4) ± SEM are shown, **p < 0.01, using ratio paired *t*-test. **B** A representative Western Blot showing levels of activated ERK (*p*-ERK1/2), ERK1/2 and as a loading control, Beta-actin. Protein lysates were from 48 h 3D acinar cultures derived from independent mice of the indicated genotype (WT, *n* = 3; *Dusp5M*^−/−^, *n* = 2). **C** Representative images and quantification (**D**) of pancreatic acinar cells cultivated in a 3D in vitro model. (Scale bars, 100 µM.) Acinar cells were derived from either wild type (WT); *n* = 7 or *Dusp5M*^−/−^; *n* = 6 mice and analysed morphologically based on the conversion of acinar cell clusters to ductal cyst structures that were comprised of a single layer of epithelial cells surrounding an empty luminal space. For each mouse and time point, the rate of transdifferentiation in four optical fields was determined. Cultures were either left untreated or stimulated by addition of epidermal growth factor (EGF; 25 ng/ml) to promote acinar cell transdifferentiation. Mean values are shown, ns not significant, **p < 0.01, ***p < 0.001, ****p < 0.0001, using unpaired *t*-test with Welch’s correction.

With respect to our conditional *Dusp5*^*fl/fl*^ strain, experiments using *Dusp5*^*fl/fl*^ MEFs indicate that this allele is hypomorphic (Supplementary Fig. S4A, B). Because DUSP5 is expressed in immune cells [35], it is possible that its loss in myeloid tissue might contribute to the pancreatic phenotype we observe. We therefore obtained a second conditional strain (*Dusp5M*^*fl/fl*^), which expresses wild type levels of DUSP5 protein and is efficiently deleted by Cre recombinase in both MEF’s and mouse tissue (Fig. S4C, D) and repeated our pancreatic cancer experiments aging mice to 56 and 100-days. Pancreas-specific deletion of *Dusp5* in this strain yields results that mirror those obtained in our original experiments with a significant increase in pancreas to body weight ratio at 56 but not 100-days (Fig. S5A, D), similar morphological changes in terms of ADM and PanIN formation (Fig. S5B, E) and progressive KRAS^G12D^-induced loss of acinar tissue (Fig. S5C, F). To further investigate a cell autonomous role for DUSP5 in regulating acinar morphology, 3D acinar cell cultures were derived from either WT or *Dusp5M*^−/−^ pancreata. In this cell culture model, KRAS^G12D^-expressing pancreatic acinar cells, or wild type pancreatic acinar cells treated with EGFR agonists, convert to ductal cysts that mimic ADM [36]. In WT acinar cells *Dusp5* mRNA levels are markedly increased over a 72 h culture period and Western blot analysis of WT and *Dusp5M*^-/−^ acinar cells after 48 h in culture reveals that levels of activated ERK are markedly increased in cells lacking DUSP5 (Fig. 3A, B). This is accompanied by a significantly increased rate of ductal cyst formation in *Dusp5M*^−/−^ acinar cells after 48 and 72 h when compared to WT, which at 48 h is enhanced by the addition of epidermal growth factor (Fig. 3C, D). Thus, even in the absence of KRAS^G12D^ expression and despite the observation that the pancreata of mice lacking DUSP5 develop normally and do not manifest signs of increased ADM (Fig. S2B), at least ex vivo DUSP5 does play a key role in the suppression of acinar transdifferentiation to a more ductal morphology.

### Loss of SERPINB2 does not influence KRAS^G12D^-driven ADM and PanIN formation in KCD5^−/−^ mice

Transgenic and knockout experiments demonstrated that SERPINB2 (plasminogen activator inhibitor-2) promotes HRAS^Q61L^-driven skin papilloma formation in mice treated with 7,12-Dimethylbenz[a]anthracene (DMBA) and 12-O-Tetradecanoylphorbol-13-acetate (TPA) [37, 38] and we demonstrated that ERK-mediated *SerpinB2* up-regulation was responsible for the elevated levels of DMBA/TPA-induced skin carcinogenesis observed in mice lacking DUSP5 [14]. Despite reports of SERPINB2 expression in human pancreatic cancers [39, 40], RT-qPCR analysis of wild type and *Dusp5*^−/−^ pancreata revealed very low levels of pancreatic *SerpinB2* mRNA expression compared with levels seen in skin (Fig. S6A). To explore the possibility that SERPINB2 overexpression caused by DUSP5 loss might promote the KRAS^G12D^-driven initiation of ADM and PanINs seen in KCD5^−/−^ pancreata, we crossed KC and KCD5^−/−^ strains with *SerpinB2* (SB2^−/−)^ knockout mice and generated age- matched cohorts of KC, KCD5^−/−^, KCSB2^−/−^ and KCD5^−/−^;SB2^−/−^ (KCDKO) animals together with *Ptf1a-Cre* controls. After 56-days, neither the KCSB2^−/−^ nor KCDKO animals displayed any significant change in pancreas to body weight ratio when compared to either KC or KCD5^−/−^ mice, respectively (Fig. S6B). Furthermore, pancreata from KCD5^−/−^ and KCDKO mice were histologically indistinguishable in terms of the extent of ADM and PanINs formation (Fig. S6C) and exhibited similar loss of acinar tissue (Fig. S6D). Finally, quantitative analysis of pancreatic lesions revealed no changes in the extent of ADM, numbers of PanINs or their progression when comparing KCD5^−/−^ and KCDKO mice (Fig. S6E, F). Thus we conclude that in contrast to results obtained in the HRAS^Q61L^-driven murine skin cancer model, SERPINB2 is not a mediator of the effects of DUSP5 loss in the pancreas.

### Loss of either DUSP5 or DUSP6 increases levels of activated ERK and expression of the ductal differentiation marker SOX9, but does not affect markers of proliferation, senescence or cell death

**Fig. 4:**
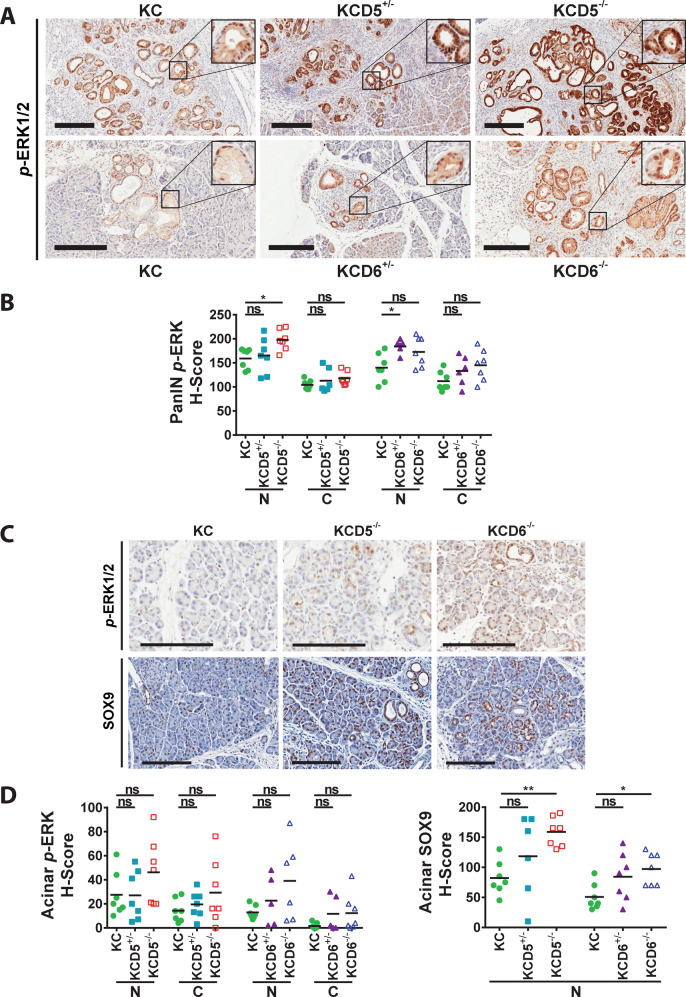
Loss of either DUSP5 or DUSP6 promotes changes in ERK activation and SOX9 expression during KRAS^G12D^-driven pancreatic carcinogenesis. Immunohistochemical analysis of 56-day age-matched pancreata of the indicated cohorts. Cohorts consisted of the following genotypes: *Kras*^*LSL-G12D/+*^*; Ptf1a-Cre; Dusp*^*+/+*^ (KC), *Kras*^*LSL-G12D/+*^*; Ptf1a-Cre; Dusp5*^*+/fl*^ (KCD5^+/−^), *Kras*^*LSL-G12D/+*^*; Ptf1a-Cre; Dusp5*^*fl/fl*^ (KCD5^−/−^), *Kras*^*LSL-G12D/+*^*; Ptf1a-Cre; Dusp6*^*+/fl*^ (KCD6^+/−^) and *Kras*^*LSL-G12D/+*^*; Ptf1a-Cre; Dusp6*^*fl/fl*^ (KCD6^−/−^). Representative images (**A**) and H-score quantification (**B**) of staining for *p*-ERK1/2 in PanINs of the indicated cohorts. Representative images (**C**) and H-score quantification (**D**) of staining for *p*-ERK1/2 and SOX9 in the acinar tissue of the indicated cohorts. (Scale bars, 200 µm.) Quantification was performed on one representative section per mouse, individual data points and mean are shown, *n* = 7 mice per cohort. N nuclear, C cytoplasmic, ns not significant, *p < 0.05, **p < 0.01, using one-way ANOVA and Bonferroni post hoc test.

DUSP5 and DUSP6 are highly specific regulators of ERK signalling [1, 3, 5]. To study the effects of deleting these phosphatases on ERK phosphorylation, tissue sections from 56-day pancreata were analysed using IHC and staining was quantified using the H-score system [41] to assess nuclear versus cytoplasmic levels of *p*-ERK. In agreement with previous work identifying DUSP5 as a specific regulator of nuclear ERK activity [14, 30], deletion of *Dusp5* caused a significant increase in levels of nuclear *p*-ERK in KRAS^G12D^-driven PanINs, but had no effect on levels of cytoplasmic ERK (Fig. 4A upper panels, B). Deletion of *Dusp6* led to modest increases in both cytoplasmic and nuclear levels of *p*-ERK (Fig. 4A lower panels, B).

When analysing acinar tissue from these sections, we also saw modest increases in *p*-ERK staining in both the nucleus and cytoplasm on loss of either DUSP5 or DUSP6 (Fig. 4C, upper panels, D, left panel). This was despite our failure to detect expression of the latter phosphatase in acinar tissue using RNAscope staining (Fig. 1E). The transcription factor SRY (sex determining region Y)-box 9 (SOX9) is involved in the specification of ductal fate during pancreatic development [42] and has been identified in acinar cells expressing KRAS^G12D^ prior to and during development of both ADM and PanINs [33]. Furthermore, while ectopic expression of SOX9 accelerates formation of KRAS^G12D^-driven premalignant lesions, genetic deletion of *Sox9* rendered the pancreas completely refractory to KRAS^G12D^-driven ADM and PanIN formation [33]. Interestingly, IHC analysis reveals that SOX9 expression is significantly elevated in the normal acinar tissue of both KCD5^−/−^ and KCD6^−/−^ pancreata compared to normal acinar tissue from KC mice (Fig. 4C, lower panels, D, right panel). This occurs independently of the greater burden of ADM and PanINs in these mice relative to KC animals. These lesions also stained positive for SOX9, but were excluded from this analysis. Acinar *p*-ERK and SOX9 staining in both KCD5^−/−^ and KCD6^−/−^ pancreata was very heterogeneous, with distinct regions of acinar tissue showing either much stronger or comparable staining to KC pancreata. This could either reflect the mosaic nature of Cre-mediated recombination in this genetic model or the fact that these clear increases in *p*-ERK and SOX9 levels are primarily occurring in acinar tissue at the onset of ADM, a process that we have shown here is clearly accelerated by the loss of either DUSP5 or DUSP6. Thus it is possible that the effects of DUSP5 or DUSP6 loss are manifest in normal acinar tissue prior to increased KRAS^G12D^-driven ADM and PanIN formation. Interestingly, SOX9 expression has been linked directly to ERK activity in both fibroblast growth factor-stimulated chondrocytes and during urothelial development, injury and carcinogenesis [43, 44] providing a possible link between loss of these ERK-specific phosphatases and expression of this driver of ADM and pancreatic malignancy. Thus SOX9, which is both essential for and can drive these neoplastic changes, could be directly up-regulated in response to deletion of either *Dusp6* or *Dusp5*. However, we cannot rule out the possibility that higher SOX9 expression is simply a marker for this process.

Finally, we also investigated other possible mechanisms underpinning the increased incidence of neoplastic changes in the pancreas on loss of DUSP5 or DUSP6 by performing IHC for markers of proliferation (Ki67), PI3-kinase-dependent survival signalling (*p*-AKT), senescence (p21 and p53) and cell death (cleaved caspase 3). Monoclonal antibody Ki67 stains cells that are actively transiting the cell cycle, but not those in G_0_ or quiescence [45]. Quantification of the average number of Ki67-positive cells within PanINs at 56-days revealed that neither loss of DUSP5 nor DUSP6 led to any significant change in the levels of this surrogate marker of proliferation, nor was any change detected in levels of signalling downstream of PI3-kinase, detected by changes in levels of *p*-AKT (Fig. S7A–D). It is well established that premalignant PanINs express many components of the senescence response, including p53, p21, p16INK4A and p19 and that tumour progression is contingent on loss or mutation of genes encoding these tumour suppressors. This suggests that senescence is triggered in response to oncogenic activation of KRAS and acts to constrain PanIN progression and tumour development [46–49]. Consistent with this, we find expression of both p53 and p21 in multiple cells across the majority of PanINs (Fig. S8A, B). However, loss of either DUSP5 or DUSP6 did not have any significant effect on the levels of these markers (Fig. S8C). Finally, we assessed levels of cleaved caspase 3, a marker of apoptosis. Consistent with previous studies, we detected only very low levels of this marker across all genotypes (Fig. S8D), with staining restricted to cells released into the lumen of PanINs and which are probably undergoing anoikis [50]. We conclude that changes in the extent of programmed cell death are unlikely to underlie any of the phenotypic changes observed on deletion of either *Dusp5* or *Dusp6*.

### Loss of DUSP6 drives the development of highly proliferative, poorly differentiated and metastatic PDAC

**Fig. 5:**
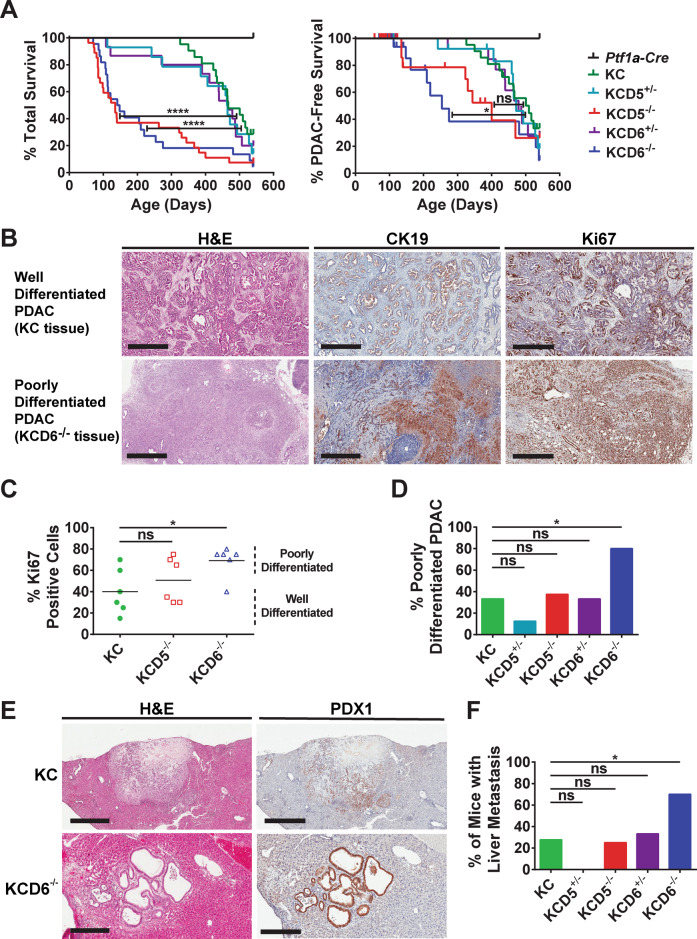
Loss of DUSP6 drives the accelerated development of highly proliferative, metastatic PDAC. **A** Kaplan–Meier curves. Cohorts consisted of the following genotypes: *Kras*^*+/+*^*; Ptf1a-Cre; Dusp*^*+/+*^ (Ptf1a-Cre), *Kras*^*LSL-G12D/+*^*; Ptf1a-Cre; Dusp*^*+/+*^ (KC), *Kras*^*LSL-G12D/+*^*; Ptf1a-Cre; Dusp5*^*+/fl*^ (KCD5^+/−^), *Kras*^*LSL-G12D/+*^*; Ptf1a-Cre; Dusp5*^*fl/fl*^ (KCD5^−/−^), *Kras*^*LSL-G12D/+*^*; Ptf1a-Cre; Dusp6*^*+/fl*^ (KCD6^+/−^) and *Kras*^*LSL-G12D/+*^*; Ptf1a-Cre; Dusp6*^*fl/fl*^ (KCD6^−/−^) Left panel, overall survival *n* = 12, 21, 14, 27, 15 and 22, respectively. Log-rank test: KC versus KCD5^−/−^ ****p < 0.0001, KC versus KCD6^−/−^ ****p < 0.0001. Right panel, PDAC-free survival *n* = 12, 0 censored; 21, 3 censored; 14, 3 censored; 27, 17 censored; 15, 3 censored and 22, 11 censored, respectively. The majority of mice were censored due to severe weight loss without PDAC, with the remainder censored due to extra-pancreatic pathologies such as skin wounds and prolapse. Log-rank test: KC versus KCD5^−/−^ ns, KC versus KCD6^−/−^ *p < 0.05. **B** Representative images of H&E staining (left panels), cytokeratin-19 IHC (CK19, middle panels) and Ki67 IHC (right panels) of either well-differentiated or poorly-differentiated PDAC tissue, taken from tumours in either KC or KCD6^−/−^ mice. (Scale bars, 500 μm.) **C** Quantification of the percentage of Ki67-positive cells in PDAC tumour sections taken from six mice of the indicated cohorts. Based on morphology, these are designated as either well- or poorly-differentiated tumours. Individual data points and mean are shown, *n* = 6, ns not significant, *p < 0.05 using one-way ANOVA and Bonferroni post hoc test. **D** Quantification of the percentage of tumour-bearing mice that displayed poorly-differentiated PDAC of the indicated cohorts. **E** Representative images of H&E staining and PDX1 IHC of liver metastases presented by KC or KCD6^−/−^ mice. (Scale bars, KC, 800 μm and KCD6^−/−^ 300 μm.) **F** The percentage of mice presenting with PDAC of the indicated cohorts that displayed associated liver metastasis. **D**, **F** *n* = 18, 8, 8, 9 and 10, ns not significant, *p < 0.05 using a 2 × 2 contingency table analysed by Fisher’s exact test with a two-tailed *p* value.

To assess the effects of loss of either DUSP5 or DUSP6 on the progression of PanIN to malignant PDAC, cohorts of KC, KCD5^+/−^, KCD5^−/−^, KCD6^+/−^, KCD6^−/−^ and *Ptf1a-Cre* controls were aged to a humane endpoint before sacrifice and examination of the pancreas and other internal organs for signs of malignant disease. Homozygous deletion of either *Dusp5* or *Dusp6* causes a significant decrease in total survival when compared to KC mice. Heterozygous loss of either phosphatase, while leading to a slightly earlier onset of mortality, has no significant effect on overall survival (Fig. 5A left panel). When the data are plotted as PDAC-free survival (Fig. 5A right panel), with cases succumbing to other pathologies censored, it is clear that homozygous loss of *Dusp6* significantly accelerates PDAC development. However, while complete deletion of *Dusp5* does result in an increase in PDAC-induced mortality, this does not reach significance overall, and heterozygous deletion of either *Dusp5* or *Dusp6* has no effect on survival. In the case of DUSP6 loss, our data were confirmed in a *Pdx1-Cre*-driven model in which *Dusp6* deletion also significantly accelerated KRAS^G12D^-driven pancreatic tumourigenesis (Fig. S9).

Poorly-differentiated PDAC displays a marked loss of glandular morphology and is associated with desmoplastic stroma, which can be visualised by staining for either cytokeratin 19 or Ki67 (Fig. 5B). Quantitation of Ki67-positive cells in PDAC from KC, KCD5^−/−^ and KCD6^−/−^ animals reveals that deletion of either phosphatase increases the level of this proliferative marker and this reached significance for *Dusp6* knockout animals. In addition, quantification of the percentage of tumour-bearing mice that displayed poorly-differentiated PDAC also shows a significant increase on loss of DUSP6 (Fig. 5C, D). Finally, metastatic spread of pancreatic tumours to the livers of affected animals is seen in KC, KCD5^−/−^ and KCD6^−/−^ mice as evidenced by lesions with ductal like morphology that exhibit positive staining for the pancreatic marker PDX1 (Fig. 5E, F). DUSP6 loss leads to a dramatically increased incidence of liver metastasis, with ~70% of KCD6^−/−^ animals with PDAC exhibiting liver metastases compared with only 28% and 25% of either KC or KCD5^−/−^ mice, respectively. Interestingly, loss of one copy of *Dusp5*, while having no effect on overall survival, does suppress both the levels of poorly-differentiated tumours and liver metastasis when compared to KC mice, though this does not reach significance (Fig. 5D, F). Heterozygous deletion of *Dusp5* also results in a decrease in acinar tissue relative to KC mice, although, again, this does not reach significance (Fig. S10E). The latter observations may thus relate to the more global health effects of DUSP5 loss (see below).

Why then are so many mice, particularly KCD5^−/−^ mice, becoming ill prior to the PDAC endpoint (Fig. S10A)? As mentioned previously, loss of either DUSP5 or DUSP6 initially causes pancreatic hyperplasia resulting in an increased pancreas to body weight ratio at 56-days (Fig. 2A). However, as mice age and ADM becomes more pronounced, the latter ratio decreases at 100-days (Fig. 2H), most probably reflecting pancreatic atrophy secondary to loss of acinar tissue (Fig. 2J). The degree of atrophy is clearly apparent compared with KC pancreata at 100-days (Supplementary Fig. S10B, C) and this reflects the almost complete loss of acinar tissue seen in these animals (Fig. S10D, E). Thus it is most likely that the outcome in terms of cause of death reflects a competitive process between weight loss due to progressive loss of functional pancreatic tissue and end-stage PDAC, although the former must be formally assessed before this conclusion is proven valid. In the case of DUSP5 loss, the extent of ADM is similar when compared with that seen on loss of DUSP6 at both 56 and 100-days However, by 100-days the number of PanINs/mm^2^ observed in KCD6^−/−^ animals is higher than that seen in mice lacking DUSP5 (Fig. 2F, K), suggesting that a greater proportion of the former mice are progressing towards PDAC. A lower progression to PDAC in mice lacking DUSP5 is also consistent with the lower levels of progression of primary PDAC to liver metastasis in this cohort (Fig. 5F).

## Discussion

Our data demonstrate that both DUSP5 and DUSP6 perform at least partially non-redundant functions in restraining the early stages of murine pancreatic cancer development driven by oncogenic mutant KRAS^G12D^. It had previously been reported that DUSP6 is up-regulated in early PanINs and that its expression is progressively lost, primarily due to epigenetic silencing, during the development of human PDAC. This, together with anti-proliferative effects of DUSP6 overexpression in human pancreatic cancer cell lines, was taken as evidence for a tumour suppressive role for DUSP6 [19–21]. Our in vivo data clearly support a key role for DUSP6 in restraining oncogenic signalling by mutant KRAS^G12D^ and suggest that its loss would both promote and accelerate the development of highly proliferative, metastatic PDAC.

In the case of DUSP5, we previously demonstrated that this phosphatase was a key regulator of nuclear ERK activity and that its deletion accelerated the development of chemically-induced HRAS^Q61L^-driven skin papillomas in mice treated with DMBA/TPA as a result of ERK-dependent up-regulation of SERPINB2 (plasminogen activator inhibitor-2) [14]. In the pancreas, we also see that DUSP5 loss acts to increase levels of both ADM and PanIN formation. Furthermore, loss of DUSP5 in cultured acinar cells both increases levels of activated ERK2 and promotes acinar transdifferentiation, a key mechanism underpinning the progression of pancreatic malignancy. These data strongly suggest a tumour suppressor role for DUSP5. The effects of DUSP5 loss were not influenced by concomitant loss of SERPINB2, indicating that this protein plays no role in promoting the formation of KRAS^G12D^-driven premalignant lesions in this tissue. Furthermore, a recent study has indicated that SERPINB2 may actually play a protective role in pancreatic cancer by restraining both aberrant remodelling of the extracellular matrix (ECM) and local invasion from primary tumours [51]. The reason for the differing role of SERPINB2 between these two tumour models is unclear. It may lie in the different Ras isoforms and/or mutations (HRAS^Q61L^ versus KRAS^G12D^) driving tumorigenesis. Tissue-specific differences in the response to DUSP5 loss may also be important, as evidenced by the lack of overlap in gene expression changes on deletion of DUSP5 in skin compared with immune cells [35]. Finally, in the PDAC model used here, KRAS^G12D^ alone drives tumourigenesis. In contrast, chemically-induced skin carcinogenesis relies on interactions between initiated cells harbouring DMBA-induced HRAS^Q61L^ mutations and inflammatory tissue hyperplasia induced by the potent tumour promoter TPA. Thus the dominant role of SERPINB2 expression in the latter model may be a result of specific tumour promotion processes in skin, which are not replicated during pancreatic cancer development. Future studies should be directed towards detailed transcriptomic and proteomic analyses of changes in pancreatic gene and protein expression/phosphorylation during KRAS^G12D^-induced malignancy in the presence and absence of DUSP5.

In terms of underlying mechanism, we find the expected increase in nuclear *p*-ERK in tissue lacking DUSP5. Modest changes in cytoplasmic and total *p*-ERK levels are also seen on deletion of *Dusp6* as manifest by a small but reproducible increase in the *p-*ERK:*p*-MEK ratio in PDAC cells derived from KCD6^−/−^ mice compared to those derived from tumours in KC animals (Fig. S11A, B). However, this does not seem to influence either cell proliferation or clonogenicity (Fig. S11C, D). Recent work has suggested that ERK-dependent phosphorylation of the mitochondrial fission GTPase Dynamin-related protein 1 (DRP1) at serine 16 is a major driver of KRAS^G12D^-driven pancreatic cancer [52, 53]. However, we have been unable to detect any significant changes in DRP1 Ser-16 phosphorylation on deletion of *Dusp6* in PDAC-derived cell lines, indicating that this may not be a relevant ERK target in the context of phosphatase loss (Fig. S11E). Finally, recent work has implicated elevated *p*-ERK activity associated with reduced expression of DUSP6 in the invasive PDAC phenotype in *LSL-Kras*^*G12D/+*^*, p53*^*fl/+*^ (KPflC) mice lacking the antioxidant protein TIGAR, further suggesting that DUSP6 loss is a factor in this reactive oxygen species-driven model of PDAC progression [54].

In conclusion, our results confirm a tumour suppressor role for both DUSP5 and DUSP6 in a clinically relevant model of mutant KRAS-driven oncogenesis. Given the propensity for loss of functional pancreatic acinar tissue secondary to ADM and atrophy on loss of either phosphatase, particularly in mice lacking DUSP5, it will be interesting to make use of the more recently developed “postnatal” models of pancreatic cancer [29] rather than the “prenatal” or developmental model used here as well as orthotopic transplantation of PDAC tumour cell lines. It would also be informative to combine the loss of DUSP5 and DUSP6 with alterations in other tumour suppressor genes known to be involved in pancreatic carcinogenesis such as *p53*, *CDKN2A* (encoding p16) or *SMAD4*. The fact that DUSP5 and DUSP6 have at least partially non-redundant functions despite regulation of a common target (ERK) in this disease model may reflect the differential regulation of ERK in either the cell nucleus or cytoplasm. However, the latter may be an oversimplification given that while DUSP5 both inactivates and anchors ERK in the nucleus, it paradoxically increases and prolongs *cytoplasmic* ERK activity [30]. Given the evidence that the RAS/ERK signalling pathway is a critical mediator of both tumour initiation and maintenance in the pancreas [55] future work should also concentrate on the identification of the critical ERK-dependent targets that are affected by loss of either DUSP5 or DUSP6.

## Materials and methods

### Experimental animals

To generate conditional *Dusp6* mice a targeting construct in which an en2A-IRESβgeoPA cassette flanked by *LoxP* sites was introduced into a Pvu1 site within intron 1 of the murine *Dusp6* gene and a third *LoxP* site tagged with a BamH1 site was introduced into a Bfr1 site downstream of the 3’ UTR was electroporated into GK 129/1 ES cells from 129 (P2) Ola mice. Following G418 selection, targeted ES cell clones were identified by Southern blotting and PCR before injection into C57BL/6J blastocysts to generate chimeric mice. The latter were then crossed with either *Pgk-Cre* animals, to remove the IRES-βgeo cassette and coding exons 2 and 3, to generate *Dusp6*^*+*/−^ mice or with *EllaCre* mice, to remove the IRES-βgeo cassette [56] and generate a conditional allele in which coding exons 2 and 3 are flanked by *LoxP* sites. Both alleles were then backcrossed through 7 generations into a C57BL/6 background. Mice targeted at the *Dusp5* or *SerpinB2* loci were as previously described [14]. A second, conditional *Dusp5* allele (*Dusp5M*) was also used, in which coding exon 1 was flanked by *LoxP* sites. Pancreas-specific expression of *Kras*^*G12D*^ and gene deletion was achieved using conditional *LSL-Kras*^G12D/+^ mice crossed with either *p48(Ptf1a)-Cre* or *Pdx-1-Cre* strains as previously described [27] and recombination was verified by PCR analysis (Fig. S12A–C). Mice were maintained under standard conditions, with free access to food and water and both male and female animals were assigned equally to cohorts after genotyping. Mice were maintained until either sacrifice at the indicated times or, for survival analysis, monitored and euthanised when the humane endpoint was reached. Sample sizes were estimated based on previous studies utilising the KC model of pancreatic cancer development, as the effect size was unknown. All mice carrying the required genotype for experimental cohorts were included in the study, with breeding halted as soon as experimental numbers were achieved. Therefore, due to the inherent genetic randomisation, no further randomisation was utilised when assigning experimental cohorts to minimise the breeding of experimental animals. Animal monitoring was performed by facility staff without knowledge of genotype. Animal work was carried out in accordance with the Animal (Scientific Procedures) Act (1986) under PPL 708570 (SMK) and PPL 7008375 (JPM) after local ethical and welfare review.

### TaqMan RT-qPCR analysis

RNA was isolated from cells and tissue using Qiashredder and RNeasy kits with on-column DNA digestion (Qiagen, Hilden, Germany) and 200 ng of RNA was reverse transcribed using TaqMan reagents (Applied Biosystems, Waltham, MA) before analysis of mRNA levels by quantitative real-time PCR using TaqMan 2x Universal Mastermix and prevalidated assay probes (Applied Biosystems, Waltham, MA) as described previously [2]. Probes used were DUSP5 (Mm01266104_m1), DUSP6 (Mm00518185_m1) and SerpinB2 (Mm00440905_m1) and levels were normalised to β-actin (Mm00607939_s1).

### Statistical analysis

For animal experiments, cohort sizes were determined as described above. All experiments in vitro were performed with three biological replicates unless otherwise stated in the figure legend. For all experiments, the appropriate statistical tests utilised are outlined in the respective figure legends. In all cases, parametric tests were utilised only when data met the assumptions of the tests, including that data is normally distributed and displays equal variance. Individual data points are plotted for all experiments with small sample sizes.

## Supplementary information

Supplemental Material
