# Supplementary material for: Suppression of mutant Kirsten-RAS (KRASG12D)-driven pancreatic carcinogenesis by dual-specificity MAP kinase phosphatases 5 and 6
Source: Oncogene. 2022 Apr 13;41(20):2811–23. doi: 10.1038/s41388-022-02302-0 (PMC9106580; doi:10.1038/s41388-022-02302-0)
Supplement: Supplementary file 1 — Supplemental Material [file 41388_2022_2302_MOESM1_ESM.pdf]

Supplementary Information for:

Suppression of mutant Kirsten-Ras ($KRAS^{G12D}$)-driven pancreatic carcinogenesis by dual-specificity MAP kinase phosphatases 5 and 6.

Andrew M. Kidger[a], Mark K. Saville[a], Linda K. Rushworth[a], Jane Davidson[a], Julia Stellzig[a], Motoharu Ono[a], Ludwig A. Kuebelsbeck[b], Klaus-Peter Janssen[b], Bernhard Holzmann[b], Jennifer P. Morton[c,d], Owen J. Sansom,[c,d], Christopher J Caunt[e] and Stephen M. Keyse[a,1]

[a]Stress Response Laboratory, Jacqui Wood Cancer Centre, Division of Cellular and Systems Medicine, School of Medicine, University of Dundee, Dundee, Scotland DD1 9SY, UK [b]Department of Surgery, School of Medicine, Klinikum rechts der Isar, Technical University of Munich, Munich, Germany. [c]Institute of Cancer Sciences, Garscube Estate, Switchback Road, Glasgow, G61 1QH. [d]CRUK Beatson Institute, Garscube Estate, Switchback Road, Glasgow, G61 1BD. [e] Department of Biology and Biochemistry, University of Bath, Claverton Down, Bath BA2 7AY, UK

Supplementary Materials and Methods

Genotyping. Genomic DNA obtained either from ear notch biopsies, cultured mouse embryo fibroblasts (MEFs) or murine PDAC cell lines was analysed by PCR for *Dusp5* and *SerpinB2* as previously described (1). For *Dusp6*, a multiplex PCR containing 5'- TTCAGACCTCATCTGAAAGACATGAGTAGT-3', 5'-CGGCGCCTGCGCCGGGGTAACCTGCCGGTGCG-3' and 5'- TGCGAGTATGA GCGCCCATTTGGTGGATGC-3' yields diagnostic PCR products of 250bp (WT), 400bp (KO) and 284bp (Floxed), respectively. For *LSL-Kras*$^{G12D/+}$, a multiplex PCR containing 5'-CCCCAGCACAGTGCAGTTTT GACACCAGCTTCGGC-3', 5'-AGCTAGCCACCATGGCTTGAGTAAGTCTGC A-3' and 5'-CCGAATTCAGTGAC TACAGATGATCAGAG-3' yields diagnostic PCR products of 450bp (WT), 484bp (*Kras*^{G12D}) and 327bp (*LSL-Kras*^{G12D}), respectively. For *Cre,* PCR reactions containing either 5'-CAAATGTTGCTTGTCTGGTG-3' and 5'-GTCAGTCGAGTG CACAGTTT-3' or 5'-ACCTGAAGATGTTCGCGATTATCT-3' and 5'-ACCGTCAG TACGTGAGAT ATCTT-3' yield diagnostic PCR products of 206 bp (Control) or 370bp (Cre), respectively. For *Dusp5M*, a multiplex PCR reaction containing 5'-CTGCACCCTGTGTTACCAGG-3', 5'-GCTGATTGATTGGATAGC-3' and 5'-CTG CAGGCAGCTCTCGGAGG-3' yields diagnostic PCR products of 711bp (WT), 959bp (Floxed) or 1047bp (KO), respectively.

Chemicals, antibodies and inhibitors. Epidermal growth factor (EGF) was from Peprotech (AF-100-15). Chemical inhibitors of MEK PD184352 (MEKi); Trametinib (GSK1120212) and PI3Kinase PI-103 (PI3Ki) were obtained from Selleck. Antibodies against *p*-ERK (#4370; #9101)*;* ERK (#4695); *p*-MEK (#9154); MEK (#9122); *p*-AKT (#4060), AKT (#4685); *p*-DRP1 (#4494); DRP1(#5391), PDX1 (#5679), cleaved caspase 3 (#9664), Ki67 (#12202) and beta-actin (#8457) were from Cell Signalling. Anti-PDX1 (Ab47267), p21 (Ab109199), α-smooth muscle actin (Ab124964) and DUSP6 (ab76310) were from Abcam. Anti-SERPINB2 (Sc-25746) was from Santa Cruz. Anti-KRAS (12063-1-AP) was from ProteinTech. Anti-β-tubulin (T8328) was from Sigma. Anti-cytokeratin19 (TROMA-III) was from DHSB Iowa. Anti-SOX9 (Ab5535) was from Millipore and anti-p53 (VP-P956) was from Vector laboratories. DUSP5 was detected using a sheep polyclonal antibody as previously described (2,3).

Western Blotting. Whole-cell lysates were prepared and analysed by SDS/PAGE using 4–12% Bis-Tris gels (Thermo Scientific, Waltham, MA) exactly as described previously (1) before quantification using primary antibodies and fluorescently tagged secondary antibodies on a Li-Cor Odyssey infrared imager using Image Studio software (Li-Cor Biosciences, Lincoln, NE). All signals shown in the Western blot panels were scanned and quantitated and phospho-epitope signals were normalised to total protein levels.

Culture of primary MEFs. Primary MEFs (3 independent lines per genotype) were isolated and cultured as described previously (1). Cell cultures were

routinely tested for mycoplasma contamination (MycoAlert #LT07-118; Lonza, Basel, CH). Transfection of MEFs with either Ad5-CMV empty or Ad5-CMVCre vectors (Vector Core, University of Iowa, USA), assays of cell proliferation, clonogenicity and analysis of *p*-ERK staining and EdU incorporation using high-content microscopy were as described previously (4). For all experiments using cultured cells technical replicates were performed in triplicate and, unless stated otherwise in the figure legends, experiments were repeated 3 times.

Tumour cell and acinar cell 3D culture. To isolate primary murine pancreatic adenocarcinoma cell lines, tumour tissue (300 mg) was diced in 0.5 ml ice cold PBS before addition of 5 ml 1 mg/mL collagenase V in DMEM. Following incubation at 37°C for 45 min, 5 ml of PDAC medium (DMEM supplemented with 20% FBS, 4.5 gl glucose, 1 mM L-glutamine, 0.11 g/l pyruvate, 50 mg/ml gentamycin, 100 U/ml penicillin and 100 mg/ml streptomycin) was added and centrifuged at 350 x g for 5 min. The partly digested tumour was then resuspended in 2 ml 0.05% trypsin/EDTA and incubated for 5 min at 37°C. The trypsin was then quenched with 2 ml PDAC medium, the solution centrifuged at 350g for 5 min and the cell pellet washed in PBS before being resuspended in 6 ml PDAC medium and seeded into a 25 cm^2 cell culture flask. After 7 days, cells were transferred into DMEM, supplemented with 10% FBS, 100 mM sodium pyruvate, 2.5 mM L-Glutamine and 100 U/ml each of Penicillin and Streptomycin and cultured for experimental use.

3D acinar cell culture was performed according to the method of Figura et al. (5) Briefly, pancreata from either wild type (WT); n = 7 or Dusp5M$^{-/-}$; n = 6 mice were isolated and digested with collagenase P (0.4 mg/ml, Roche 11213857001) in HBSS for 10 min at 37 °C with gentle agitation. Digestion was then stopped by the addition of 5% FBS/HBSS. The cells were washed twice with 5% FBS/HBSS and filtered through a 100 µm cell strainer. The cell suspension was layered over a 30% FBS/HBSS solution to allow intact cell clusters to precipitate by low-speed centrifugation at 180 x g. The acinar cell clusters were resuspended in growth factor reduced Matrigel (BD 354230) with an equal volume of 3D culture media (Waymouth's media, 10% FBS, 100 U/ml penicillin, 100 µg/ml streptomycin, 2 mM L-glutamine, 100 µg/ml soybean trypsin inhibitor, 1 µg/ml dexamethasone) and seeded into rat-tail collagen-1 (BD 354236) coated wells of a 24-well plate. Cells were then covered with an additional layer of collagen-1 and 3D culture media was added. The transdifferentiation process was evaluated morphologically over time, based on the conversion of acinar cell clusters to ductal cyst structures that were comprised of a single layer of epithelial cells surrounding an empty luminal space. For each animal and time point, the mean rate of transdifferentiation in 4 optical fields was determined.

Tissue sectioning and histology. Organs were fixed, embedded, sectioned and stained with H & E or analysed by immunohistochemical (IHC) staining as previously described (1). Quantitative analysis of all H & E and IHC staining was performed blinded. Alcian blue staining was performed following rehydration for 30 min in Alcian blue solution (Sigma) before counterstaining with Nuclear Fast

Red (Sigma) for 5 min. Slides were then dehydrated through increasing concentrations of ethanol (70%, 90%, 95% & 100% x 2 - 2 min each) and cleared in xylenes (2 x 10 min) before mounting under coverslips using Omnimount (National Diagnostics, Atlanta, GA). All slides required for imaging or quantitative analysis were scanned on a Leica Biosystems Aperio XT slide scanner and quantitative analysis was performed using Aperio ImageScope software (Leica Biosystems, Wetzlar, Germany).

RNAscope analysis. *In situ hybridisation* detection of *Dusp6* (Advanced Cell Diagnostics, Hayward, CA; 429328) mRNA was performed using RNAscope 2.5 LS (Brown) detection kit (Advanced Cell Diagnostics, Hayward, CA; 322100) on a Leica Bond Rx autostainer according to the manufacturer's instructions Leica Biosystems, Wetzlar, Germany). Staining was performed on 4um formalin-fixed paraffin-embedded sections, which were cut and placed in a 60°C oven 2 h prior to staining. In order to verify RNA integrity in the tissue, a positive control probe (mm-Ppib, 313918) was used.

References

1. Rushworth LK, Kidger AM, Delavaine, L, Stewart G, van Schelven S, Davidson J, et al. Dual-specificity phosphatase 5 regulates nuclear ERK activity and suppresses skin cancer by inhibiting mutant Harvey-Ras (HRasQ61L)-driven SerpinB2 expression. Proceedings of the National Academy of Sciences of the United States of America 2014;111: 18267-72
2. Mandl M, Slack DN, & Keyse SM, Specific inactivation and nuclear anchoring of extracellular signal-regulated kinase 2 by the inducible dual-specificity protein phosphatase DUSP5. Molecular and Cellular Biology. 2005;25: 1830-45.
3. Kucharska A, Rushworth LK, Staples C, Morrice NA, & Keyse SM, Regulation of the inducible nuclear dual-specificity phosphatase DUSP5 by ERK MAPK. Cellular Signalling. 2009; 21: 1794-805 (2009).
4. Kidger AM, Rushworth, LK, Stellzig J, Davidson J, Bryant CJ, Bayley C, et al. Dual-specificity phosphatase 5 controls the localized inhibition, propagation, and transforming potential of ERK signaling. Proceedings of the National Academy of Sciences of the United States of America. 2017;114:E317-E26.
5. von Figura G, Morris JP 4th, Wright CV, Hebrok M, Nr5a2 maintains acinar cell differentiation and constrains oncogenic Kras-mediated pancreatic neoplastic initiation. Gut. 2014; 63: 656-64.

SI Figure legends

Fig. S1. Up-regulation of DUSP5 and DUSP6 in response to endogenous $KRAS^{G12D}$ is ERK1/2-dependent. (*A-C*) *Kras*$^{LSL\text{-}G12D/+}$ MEFs were infected with either empty adenovirus (Ad5-Empty) or adenoviral-*Cre* (Ad5-Cre) for 44 h prior to treatment with either DMSO, the MEK inhibitor PD184352 (MEKi, 10 μM) or the PI3K inhibitor Pi103 (PI3Ki, 1 μM) for 4 h prior to cell lysis, and either RNA isolation (*A*) or analysis by immunoblotting using the indicated antibodies (*B, C*). The experiment was performed 3 times using independent MEF lines for each genotype (*A*) TaqMan RT-qPCR analysis of the indicated transcripts showing the fold change in mRNA levels relative to vehicle (DMSO) treated cells infected with empty adenovirus. (*B*) A Western blot representative of 3 independent experiments is shown, alongside graphs (*C*) showing the fold change in protein levels relative to vehicle (DMSO) treated cells infected with empty adenovirus. The tubulin blot is shown as a representative loading control (*p*-ERK1/2/ERK1/2). For all quantitative data individual experimental points and mean values from 3 independent experiments (n = 3) ± SEM are shown, ns = not significant, *P < 0.05, **P < 0.01, ***P < 0.001, ****P < 0.0001 using one-way ANOVA and Bonferroni post hoc test.

Fig. S2. Loss of either DUSP5 or DUSP6 does not alter normal pancreatic development. (*A*) Pancreata from age-matched 5-month-old mice (n = 2) of the indicated cohorts were harvested and their pancreas to body weight ratios calculated. *Kras*$^{LSL\text{-}G12D/+}$; *Ptf1a-Cre* (KC) mice were included as positive controls for pancreatic tumourigenesis. (*B*) Representative H&E stained images of wild type (WT) or knockout (either *Dusp5*$^{-/-}$ or *Dusp6*$^{-/-}$) pancreata isolated from age-matched 5-month-old mice. Asterisks indicate Islets of Langerhans and arrows indicate pancreatic ducts. (Scale bars, 200 μm.)

Fig. S3. Loss of either DUSP6 or DUSP6 promotes increased $KRAS^{G12D}$-driven initiation of pancreatic carcinogenesis. Representative images of H&E stained pancreata from 56-day (*A*) or 100-day (*B*) age-matched mice of the indicated cohorts. *Kras*$^{+/+}$; *Ptf1a-Cre*; *Dusp*$^{+/+}$ (Ptf1a-Cre), *Kras*$^{LSL\text{-}G12D/+}$*; Ptf1a-Cre; Dusp*$^{+/+}$ (KC), *Kras*$^{LSL\text{-}G12D/+}$*; Ptf1a-Cre; Dusp5*$^{+/fl}$ ($KCD5^{+/-}$), *Kras*$^{LSL\text{-}G12D/+}$*; Ptf1a-Cre; Dusp5*$^{fl/fl}$ ($KCD5^{-/-}$), *KRas*$^{LSL\text{-}G12D/+}$*; Ptf1a-Cre; Dusp6*$^{+/fl}$ ($KCD6^{+/-}$) and *Kras*$^{LSLG12D/+}$*; Ptf1a-Cre; Dusp6*$^{fl/fl}$ ($KCD6^{-/-}$). (Scale bars, 1mm.)

Fig. S4. Validation of a true-conditional *Dusp5M* allele. (*A*) TaqMan RT-qPCR assay showing the fold change in *Dusp5* mRNA levels of *Dusp5*$^{+/+}$*, Dusp5*$^{fl/fl}$ and *Dusp5*$^{-/-}$ MEFs following TPA stimulation (100 ng/ml) relative to the unstimulated *Dusp5*$^{+/+}$ MEFs. The result of 2 experiments performed using independent cell lines of the indicated genotypes with mean values is shown. (*B*) MEFs of the indicated genotype were stimulated with TPA for 0, 1, 2 & 4 h prior to cell lysis, and analysis by immunoblotting using the indicated antibodies. A representative Western blot is shown and the tubulin blot is included as a representative loading control (*p*-ERK1/2/ERK1/2) (*C*) MEFs of the indicated genotype for the true

conditional allele *Dusp5M* were infected with either empty adenovirus (Ad5-Empty) or adenoviral-*Cre* (Ad5-Cre) for 48 h prior to treatment with TPA for 0 & 2 h before cell lysis, and analysis by immunoblotting using the indicated antibodies. The tubulin blot is shown as a loading control (*D*) TaqMan RT-qPCR assay showing levels of *Dusp5* and *Dusp6* mRNA expression in the thymus from each of 3 animals of the true conditional (*Dusp5M*$^{fl/fl}$) strain before and after crossing with *Cre*-deleter mice. Mean values ± SEM are shown, n = 3.

Fig. S5. The conditional, pancreas-specific loss of *Dusp5M* promotes increased KRAS^{G12D}-driven initiation of pancreatic carcinogenesis. Pancreata from 56- (*A*) or 100-day (*D*) age-matched mice of the indicated cohorts were harvested and their pancreas to body weight ratios calculated. Cohorts consisted of the following genotypes: *Kras*$^{+/+}$*; Ptf1a-Cre; Dusp*$^{+/+}$ (Ptf1a-Cre), *Kras*$^{LSL\text{-}G12D/+}$; *Ptf1a-Cre; Dusp*$^{+/+}$ (KC), *Kras*$^{LSL\text{-}G12D/+}$*; Ptf1a-Cre; Dusp5M*$^{+/fl}$ (KCD5M$^{+/-}$), *Kras*$^{LSL\text{-}G12D/+}$*; Ptf1a-Cre; Dusp5M*$^{fl/fl}$ (KCD5M$^{-/-}$). Mean values are shown, n = 3-7 mice per cohort. ns = not significant, *P < 0.05, using one-way ANOVA and Bonferroni post hoc test. Representative images of H&E stained pancreata from 56- (*B*) or 100-day (*E*) age-matched mice of the indicated cohorts. (Scale bars, 200 µm.) Quantification of the percentage acinar tissue remaining in either 56 (*C*) or 100-day (*F*) age-matched pancreata of the indicated cohorts following KRAS^{G12D}-driven ADM and PanIN initiation. Quantification was performed on one representative section per mouse, following serial sectioning of the pancreas. Mean values are shown, n = 7 mice per cohort, ns = not significant, **P < 0.01, ****P < 0.0001, using one-way ANOVA and Bonferroni post hoc test.

Fig. S6. Loss of SERPINB2 does not influence KRAS^{G12D}-driven ADM and PanIN formation in KCD5$^{-/-}$ mice. (*A*) TaqMan RT-qPCR analysis showing mRNA levels of *SerpinB2*, relative to Beta-actin (*Actb*), following RNA isolation from the skin and pancreata of two 100-day age-matched mice of the indicated genotype. Mean values are shown (n = 2). (*B*) Pancreata from 56-day age-matched mice of the indicated cohorts were harvested and their pancreas to body weight ratios calculated. Cohorts consisted of the following genotypes *Kras*$^{+/+}$; *Ptf1a-Cre*; *Dusp5*$^{+/+}$; *SerpinB2*$^{+/+}$ (Ptf1a-Cre), *Kras*$^{LSL\text{-}G12D/+}$*; Ptf1a-Cre; Dusp*$^{+/+}$*; SerpinB2*$^{+/+}$ (KC), *Kras*$^{LSL\text{-}G12D/+}$*; Ptf1a-Cre; Dusp5*$^{+/fl}$*; SerpinB2*$^{+/+}$ (KCD5$^{+/-}$), *Kras*$^{LSL\text{-}G12D/+}$*; Ptf1a-Cre; Dusp5*$^{fl/fl}$; SerpinB2$^{+/+}$(KCD5$^{-/-}$), *Kras*$^{LSL\text{-}G12D/+}$*; Ptf1a-Cre; Dusp5*$^{+/+}$*; SerpinB2*$^{-/-}$ (KCSB2$^{-/-}$) and *Kras*$^{LSL\text{-}G12D/+}$*; Ptf1a-Cre; Dusp5*$^{fl/fl}$*; SerpinB2*$^{-/-}$ (KCDKO). Mean values are shown, n = 5, 7, 7, 7, 7 and 7 mice per cohort, ns = not significant, *P < 0.05, ****P < 0.0001 using one-way ANOVA and Bonferroni post hoc test. (*C*) Representative images of H & E stained pancreata from 56-day age-matched mice of the indicated cohorts. (Scale bars, 200 µm.) (*D-F*) Quantification of pancreatic lesion precursor development in 56-day age-matched pancreata of the indicated cohorts. (*D*) Percentage of acinar tissue remaining in the pancreata of each cohort following KRAS^{G12D}-driven ADM and PanIN initiation. (*E*) Total number of PanIN's of all histological grades per mm^2 in the indicated cohorts. (*F*) Quantification of the number of pancreatic precursor lesions, divided into each histological grade, expressed as a percentage of the

total number of lesions per cohort. Quantification was performed on one representative section per mouse, following serial sectioning of the pancreas. Mean values ± SEM are shown, n = 7 mice per cohort, ns = not significant, *P < 0.05, **P < 0.01, ***P < 0.001, ****P < 0.0001 using one-way ANOVA and Bonferroni post hoc test. Please note that due to concomitant recruitment of both the KCSB2$^{-/-}$ and KCDKO mice into the aged cohorts the quantitative data pertaining to outcomes in KC, KCD5$^{+/-}$ and KCD5$^{-/-}$ mice shown in *D, E* and *F* is the same as that presented in Figure 2 (*E-G*).

Fig. S7. Loss of either DUSP5 or DUSP6 does not alter levels of either Ki67 or *p*-AKT during KRAS^{G12D}-driven pancreatic carcinogenesis. Immunohistochemical analysis of 56-day age-matched pancreata of the indicated cohorts. Cohorts consisted of the following genotypes: *Kras$^{LSL-G12D/+}$; Ptf1a-Cre; Dusp$^{+/+}$* (KC), *Kras$^{LSL-G12D/+}$; Ptf1a-Cre; Dusp5$^{+/fl}$* (KCD5$^{+/-}$), *Kras$^{LSL-G12D/+}$; Ptf1a-Cre; Dusp5$^{fl/fl}$* (KCD5$^{-/-}$), *Kras$^{LSL-G12D/+}$; Ptf1a-Cre; Dusp6$^{+/fl}$* (KCD6$^{+/-}$) and *Kras$^{LSL-G12D/+}$; Ptf1a-Cre; Dusp6$^{fl/fl}$* (KCD6$^{-/-}$). (*A-B*) Representative images of staining for Ki67 (*A*) and quantification of the percentage of Ki67-positive cells per PanIN (*B*) of the indicated cohorts. (*C-D*) Representative images (*C*) and H-score quantification (*D*) of staining for *p*-AKT in PanINs of the indicated cohorts. (Scale bars, 200μm.) Quantification was performed on one representative section per mouse, individual data points and mean values are shown, n = 7, N = nuclear, C = cytoplasmic, ns = not significant, using one-way ANOVA and Bonferroni post hoc test.

Fig. S8. Loss of either DUSP5 or DUSP6 does not alter markers of senescence or cell death during KRAS^{G12D}-driven pancreatic carcinogenesis. Immunohistochemical analysis of 56-day age-matched pancreata of the indicated cohorts. Cohorts consisted of the following genotypes: *Kras$^{LSL-G12D/+}$; Ptf1a-Cre; Dusp$^{+/+}$* (KC), *Kras$^{LSL-G12D/+}$; Ptf1a-Cre; Dusp5$^{+/fl}$* (KCD5$^{+/-}$), *KrasL$^{SL-G12D/+}$; Ptf1a-Cre; Dusp5$^{fl/fl}$* (KCD5$^{-/-}$), *Kras$^{LSLG12D/+}$; Ptf1a-Cre; Dusp6$^{+/fl}$* (KCD6$^{+/-}$) and *Kras$^{LSLG12D/+}$; Ptf1a-Cre; Dusp6$^{fl/fl}$* (KCD6$^{-/-}$). Representative images of staining for p53 (*A*), p21 (*B*) and cleaved caspase-3 (*D*). (Scale bars, 200μm.) (*C*) H-score quantification of staining for p53 and p21 in PanINs of the indicated cohorts. Quantification was performed on one representative section per mouse, individual data points and mean values are shown, n = 7, N = nuclear, C = cytoplasmic, ns = not significant, using one-way ANOVA and Bonferroni post hoc test.

Fig. S9. DUSP6 loss drives the accelerated development of PDAC. Kaplan-Meier curves showing PDAC-free survival of the indicated cohorts. Cohorts consisted of the following genotypes *Kras$^{LSL-G12D/+}$; Pdx1-Cre; Dusp6$^{+/+}$* (KC, n = 49, 38 censored), *Kras$^{LSL-G12D/+}$; Pdx1-Cre; Dusp6$^{+/-}$* (KCD6$^{+/-}$, n = 53, 32 censored) and *Kras$^{LSL-G12D/+}$; Pdx1-Cre; Dusp6$^{-/-}$* (KCD6$^{-/-}$, n = 63, 42 censored). Mice were censored due to extra-pancreatic pathologies as previously reported in this model, predominantly skin papillomas, lymphomas and intussusceptions.

Logrank test, ns = not significant, **P < 0.01, ***P < 0.001.

Fig. S10. Loss of either DUSP5 or DUSP6 in the presence of KRAS^{G12D} frequently promotes extensive PanIN formation and the complete loss of acinar tissue. (*A*) The percentage of mice of the indicated cohorts sacrificed due to ill health, typically dramatic weight-loss (WL), which upon dissection did not present with PDAC. Cohorts consisted of the following genotypes: *Kras$^{LSL\text{-}G12D/+}$; Ptf1a-Cre; Dusp$^{+/+}$* (KC, n = 21), *Kras$^{LSL\text{-}G12D/+}$; Ptf1a-Cre; Dusp5$^{+/fl}$* (KCD5$^{+/-}$, n = 14), *Kras$^{LSL\text{-}G12D/+}$; Ptf1a-Cre; Dusp5$^{fl/fl}$* (KCD5$^{-/-}$, n = 27), *Kras$^{LSL\text{-}G12D/+}$; Ptf1a-Cre; Dusp6$^{+/fl}$* (KCD6$^{+/-}$, n = 15) and *Kras$^{LSL\text{-}G12D/+}$; Ptf1a-Cre; Dusp6$^{fl/fl}$* (KCD6$^{-/-}$, n = 22), ns = not significant, **P < 0.01,****P < 0.0001 using a 2 x 2 contingency table analysed by Fisher's exact test with a two-tailed P value. (*B*) Images of isolated pancreata from 2 mice of the indicated genotype demonstrating the reduction in size of KCD5$^{-/-}$ pancreata, relative to KC. (*C*) Quantification of the pancreas to body weight ratio of the indicated cohorts. The KCD5$^{-/-}$ and KCD6$^{-/-}$ WL cohorts represent all the mice of these genotypes that were included in the survival study, but which had to be removed following dramatic weight-loss, and did not present with PDAC. KCD5$^{-/-}$ WL cohort median age, 94 days; KCD6$^{-/-}$ WL cohort median age, 113 days. Mean shown, n = 5, 4, 6, 9, 6, 7, 10, 7, 7, 9. (*D*) A representative image of an H&E stained pancreatic tissue section from a KCD5$^{-/-}$ WL cohort animal. (Scale bars, 2mm.) (*E*) Quantification of the percentage acinar tissue remaining in the pancreata of the indicated 100-day age-matched cohorts alongside the KCD5$^{-/-}$ WL and KCD6$^{-/-}$ WL cohorts. Mean shown, n = 9, 7, 7, 12, 7, 8, 7, ns = not significant, **P < 0.01, ***P < 0.001,****P < 0.0001, using one-way ANOVA and Bonferroni post hoc test.

Fig. S11. Characterisation of the effects of DUSP6 loss in KRAS^{G12D}-driven PDAC cell lines. (*A-B*) PDAC cell lines isolated from individual mice with tumours from either the *Kras$^{LSL\text{-}G12D/+}$; Ptf1a-Cre; Dusp$^{+/+}$* (**KC,** n = 6) or *Kras$^{LSL\text{-}G12D/+}$; Ptf1a-Cre; Dusp6$^{fl/fl}$* (KCD6$^{-/-}$, n = 4) cohorts were lysed and immunoblotted using the indicated antibodies. A representative Western blot from 3 independent experiments is shown (*A*), alongside protein quantification of the *p*-ERK: *p*-MEK ratio (*B*). The tubulin blot is shown as a representative loading control (*p*-ERK1/2/ERK1/2). Data points represent the mean *p*-ERK: *p*-MEK ratio across the 3 independent experiments for each of the individual PDAC cell lines. Mean of each cohort shown (KC, n = 6; KCD6$^{-/-}$, n = 4). **P < 0.01, using an unpaired t-test. (*C*) KC (n = 6) or KCD6$^{-/-}$ (n = 4) cell lines were maintained at the indicated percentage serum and proliferation assessed by real-time imaging in 3-4 independent experiments at 72 h. Values are the mean for each cohort ± SEM, n = 4-6. (*D*) KC (n = 6) or KCD6$^{-/-}$ (n = 4) cell lines were maintained at the indicated serum percentage and colony formation evaluated in 3-4 independent experiments. Values are the mean for each cohort ± SEM, n = 4-6. (*E*) KC (n = 6) or KCD6$^{-/-}$ (n = 4) PDAC cell lines were lysed and analysed by immunoblotting using the indicated antibodies. A representative Western blot from 3 independent experiments is shown and a tubulin blot is shown as a representative loading control (*p*-ERK1/2/ERK1/2).

Fig. S12. Validation of *Ptf1a-Cre* mediated recombination of conditional alleles in the pancreas. (*A*) Gel electrophoresis visualisation of the PCR products for pancreatic genomic DNA from *Kras*$^{LSL-G12D/+}$; *Ptf1a-Cre* mice. The mosaic excision of the Lox-STOP-Lox (LSL)-cassette from the *Kras*$^{LSL-G12D}$ allele in the presence of *Ptf1a-Cre* expression in the pancreas can be observed (lanes 1 and 2). Cre-mediated recombination of the *Kras*$^{LSL-G12D}$ allele in MEFs results in the formation of a 484 bp fragment (LSL+Cre, lane 4) while lysates from untreated *Kras*$^{LSL-G12D/LSL-G12D}$ (LSL, lane 5) and *Kras*$^{+/+}$ (WT, lane 6) MEFs validate the 327 bp LSL-cassette and 450 bp WT bands, respectively. The no template PCR control is shown in lane 3. (*B*) Gel electrophoresis visualisation of the PCR products for pancreatic genomic DNA from *Ptf1a-Cre; Dusp5*$^{fl/fl}$ mice demonstrating the excision of exon 2 of the *Dusp5* allele in the presence of *Ptf1a-Cre* (Lanes 1 and 2). Control ear notch DNA samples from *Dusp5*$^{fl/fl}$ (Lox, lane 3), *Dusp5*$^{-/-}$ (KO, lane 4) and *Dusp5*$^{+/+}$ (WT, lane 5) mice validate the 701 bp, 418 bp and 667 bp bands, respectively. The no template PCR control is shown in lane 3. (*C*) Gel electrophoresis visualisation of the PCR products produced for pancreatic genomic DNA from *Ptf1a-Cre; Dusp6*$^{fl/fl}$ mice demonstrating the excision of *Dusp6* exons 2 and 3 in the presence of *Ptf1a-Cre* (lanes 1-4). Control ear notch DNA samples from *Dusp6*$^{fl/fl}$ (Lox, lane 6) and *Dusp6*$^{-/-}$ (KO, lane 7) mice validate the 284 bp and 400 bp bands, respectively. The no template PCR control is shown in lane 5**.**

Fig. S1

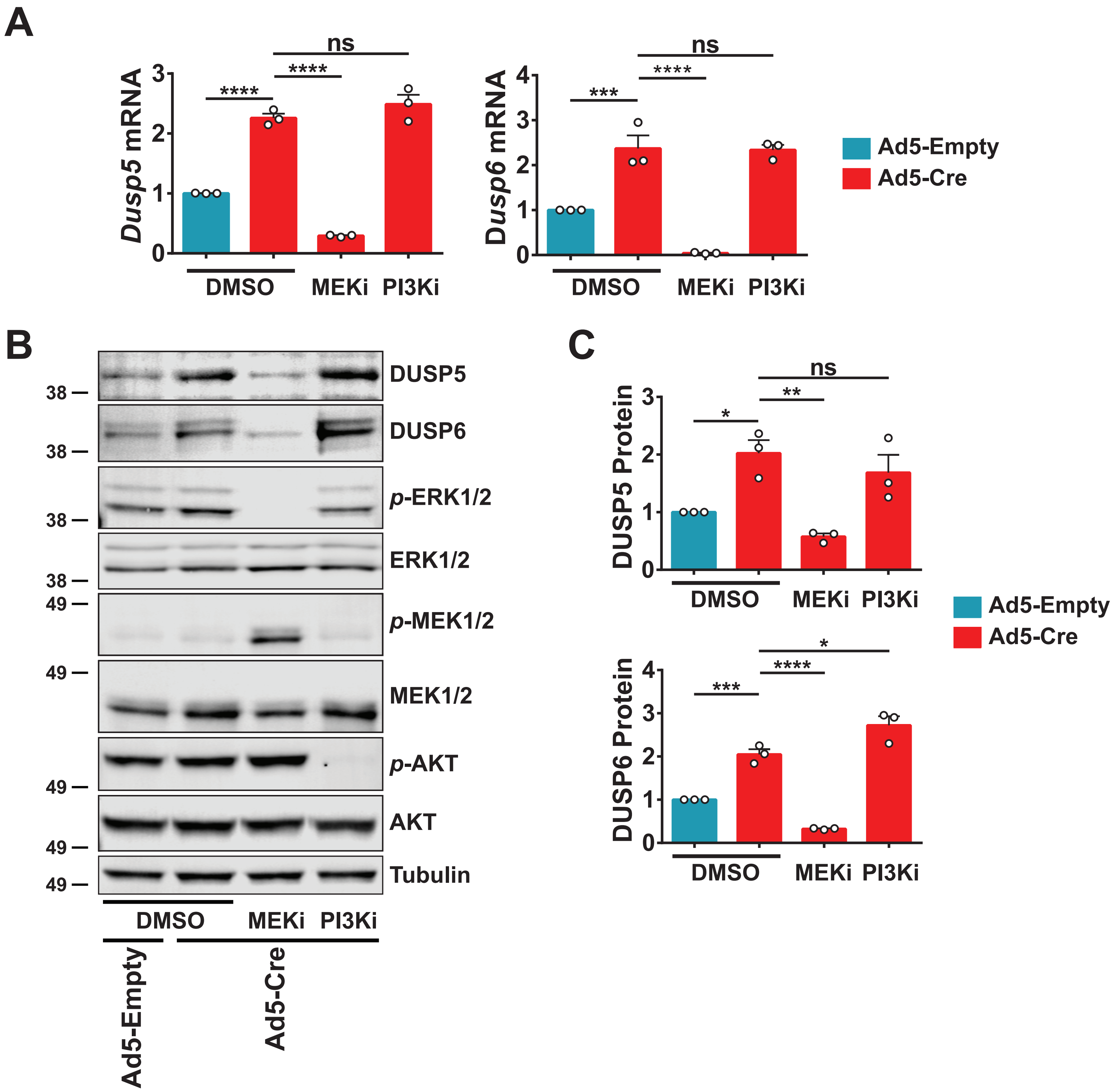
A
Dusp5 mRNA
Dusp6 mRNA
ns
****
***
DMSO
MEKi
PI3Ki
Ad5-Empty
Ad5-Cre
B
DUSP5
DUSP6
p-ERK1/2
ERK1/2
p-MEK1/2
MEK1/2
p-AKT
AKT
Tubulin
38
49
DMSO
MEKi
PI3Ki
Ad5-Empty
Ad5-Cre
C
DUSP5 Protein
DUSP6 Protein
ns
**
*
****
***
DMSO
MEKi
PI3Ki
Ad5-Empty
Ad5-Cre

Fig. S2

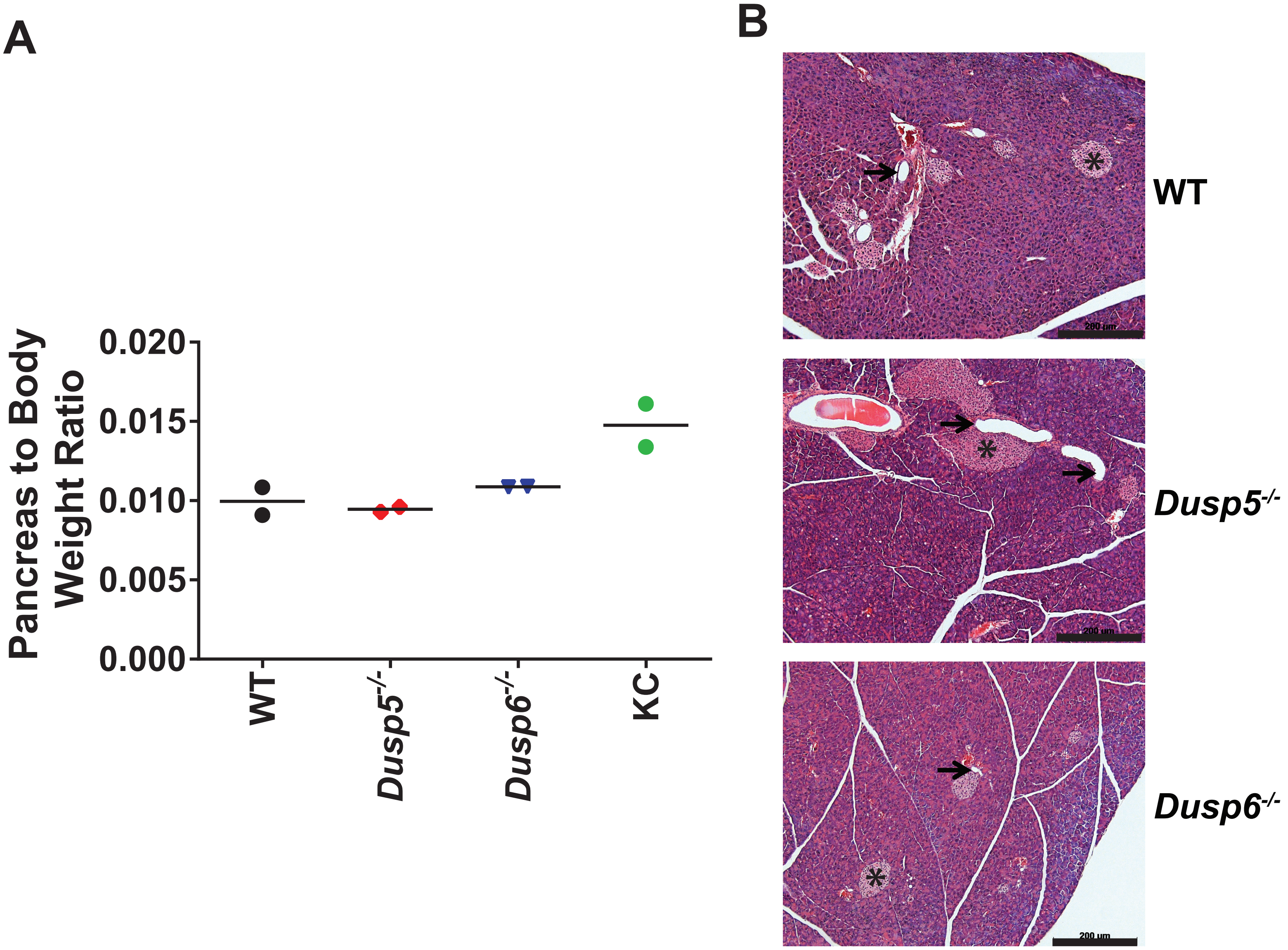
A
Pancreas to Body Weight Ratio
0.020
0.015
0.010
0.005
0.000
WT
Dusp5-/-
Dusp6-/-
KC
B
WT
Dusp5-/-
Dusp6-/-
200 μm

Fig. S3

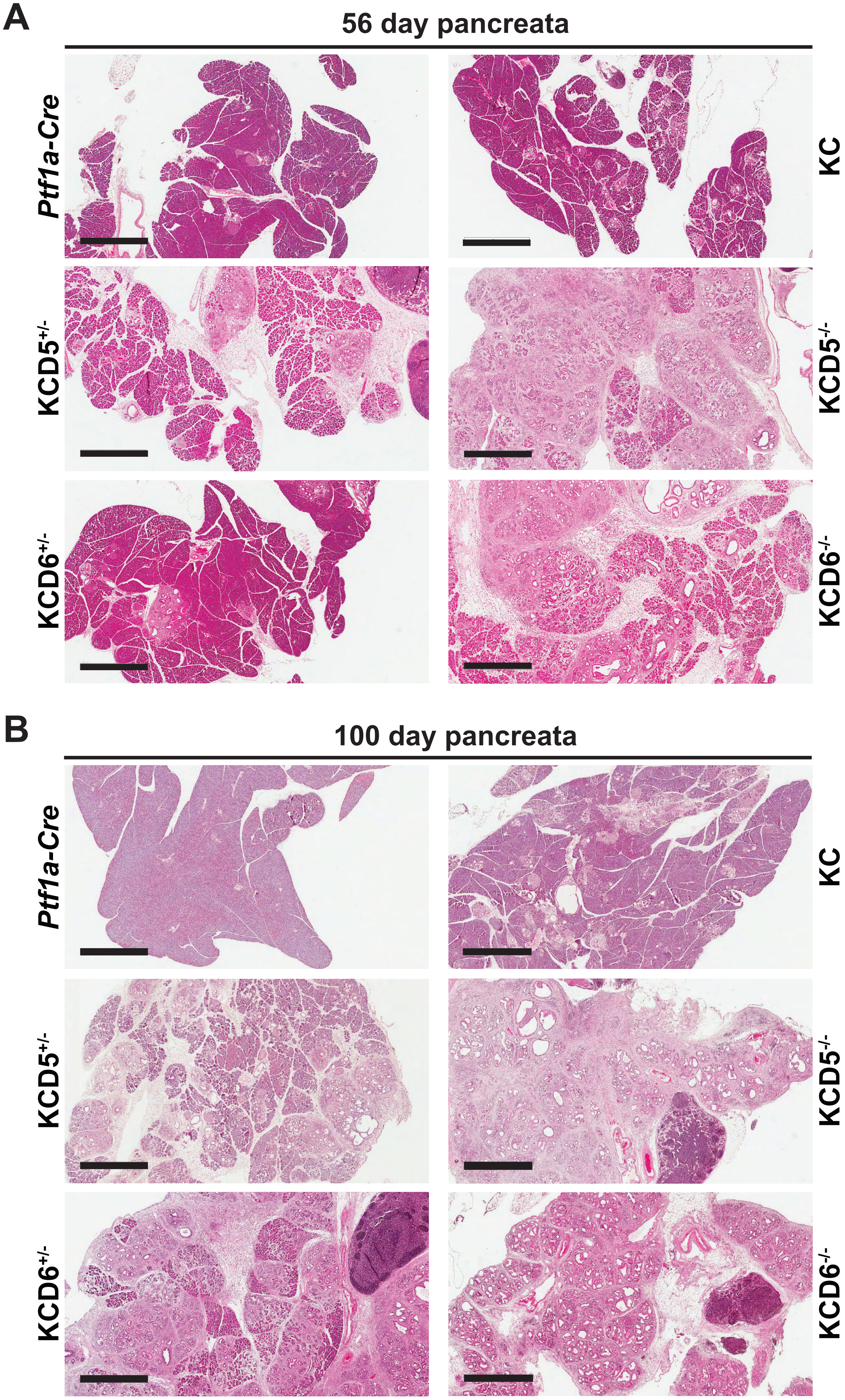
A
56 day pancreata
Ptf1a-Cre
KC
KCD5+/-
KCD5-/-
KCD6+/-
KCD6-/-
B
100 day pancreata
Ptf1a-Cre
KC
KCD5+/-
KCD5-/-
KCD6+/-
KCD6-/-

Fig. S4

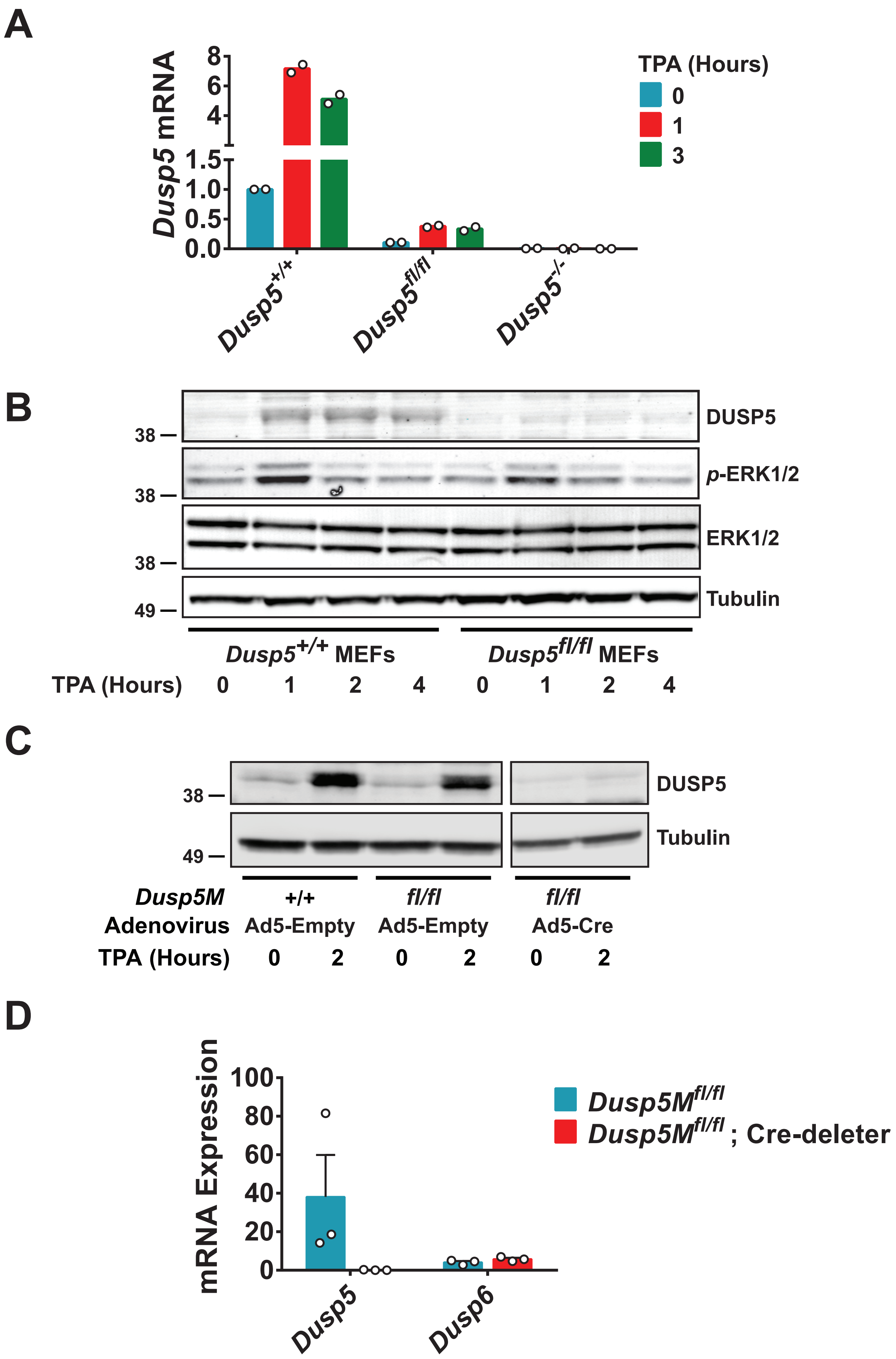
A
Dusp5 mRNA
8
6
4
1.5
1.0
0.5
0.0
TPA (Hours)
0
1
3
Dusp5+/+
Dusp5fl/fl
Dusp5-/-
B
DUSP5
p-ERK1/2
ERK1/2
Tubulin
38
38
38
49
Dusp5+/+ MEFs
Dusp5fl/fl MEFs
TPA (Hours) 0 1 2 4 0 1 2 4
C
DUSP5
Tubulin
38
49
Dusp5M +/+ fl/fl fl/fl
Adenovirus Ad5-Empty Ad5-Empty Ad5-Cre
TPA (Hours) 0 2 0 2 0 2
D
mRNA Expression
100
80
60
40
20
0
Dusp5
Dusp6
Dusp5Mfl/fl
Dusp5Mfl/fl ; Cre-deleter

Fig. S5

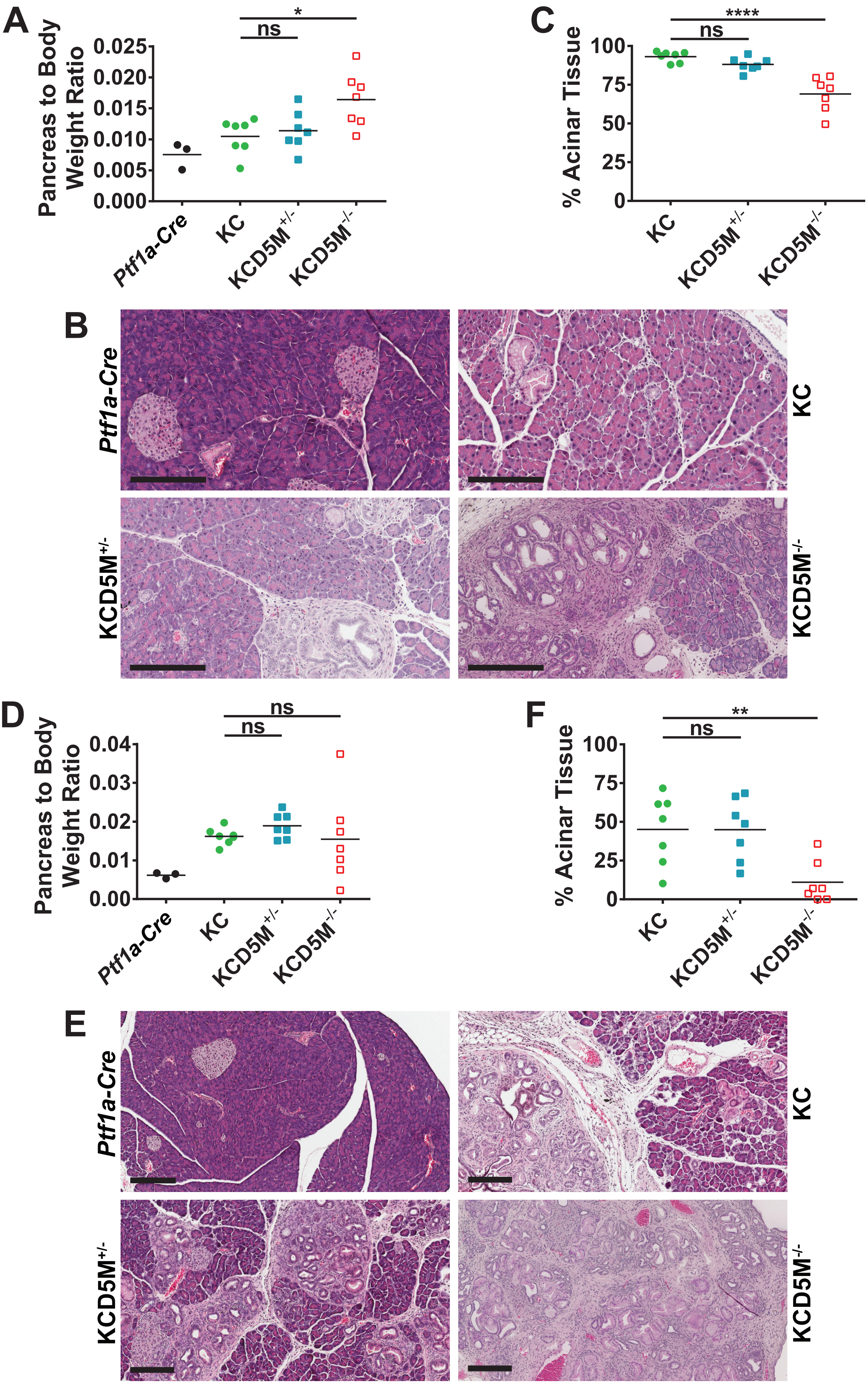

A
Pancreas to Body Weight Ratio
0.025
0.020
0.015
0.010
0.005
0.000
ns
*
Ptf1a-Cre
KC
KCD5M+/-
KCD5M-/-
C
% Acinar Tissue
100
75
50
25
0
ns
****
KC
KCD5M+/-
KCD5M-/-
B
Ptf1a-Cre
KC
KCD5M+/-
KCD5M-/-
D
Pancreas to Body Weight Ratio
0.04
0.03
0.02
0.01
0.00
ns
ns
Ptf1a-Cre
KC
KCD5M+/-
KCD5M-/-
F
% Acinar Tissue
100
75
50
25
0
ns
**
KC
KCD5M+/-
KCD5M-/-
E
Ptf1a-Cre
KC
KCD5M+/-
KCD5M-/-

Fig. S6

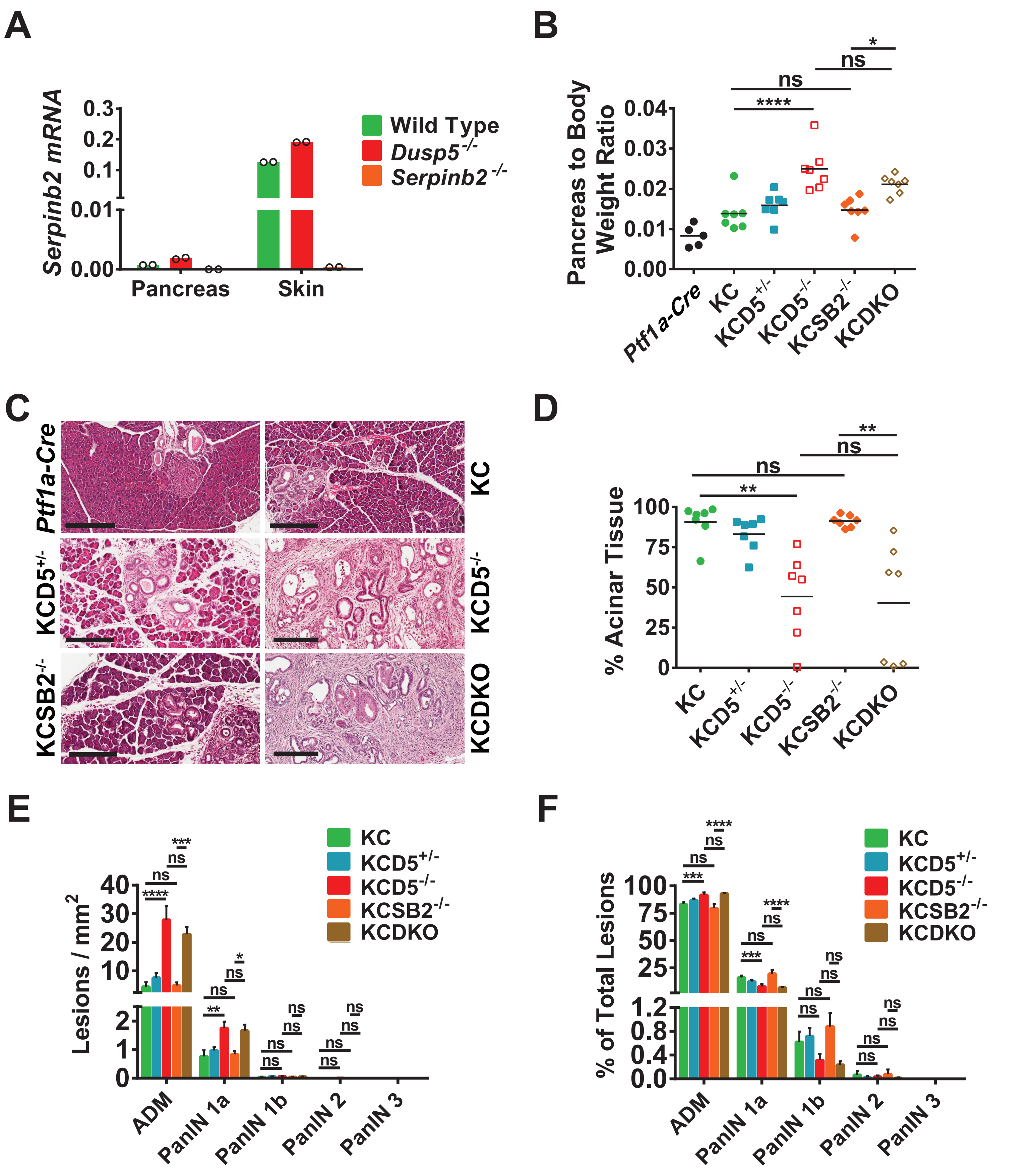
A
Serpinb2 mRNA
0.3
0.2
0.1
0.01
0.00
Pancreas
Skin
Wild Type
Dusp5-/-
Serpinb2-/-
B
Pancreas to Body Weight Ratio
0.04
0.03
0.02
0.01
0.00
Ptf1a-Cre
KC
KCD5+/-
KCD5-/-
KCSB2-/-
KCDKO
ns
****
ns
*
C
Ptf1a-Cre
KC
KCD5+/-
KCD5-/-
KCSB2-/-
KCDKO
D
% Acinar Tissue
100
75
50
25
0
KC
KCD5+/-
KCD5-/-
KCSB2-/-
KCDKO
**
ns
ns
**
E
Lesions / mm2
40
30
20
10
2
1
0
ADM
PanIN 1a
PanIN 1b
PanIN 2
PanIN 3
KC
KCD5+/-
KCD5-/-
KCSB2-/-
KCDKO
F
% of Total Lesions
100
75
50
25
1.2
0.8
0.4
0.0
ADM
PanIN 1a
PanIN 1b
PanIN 2
PanIN 3
KC
KCD5+/-
KCD5-/-
KCSB2-/-
KCDKO

Fig. S7

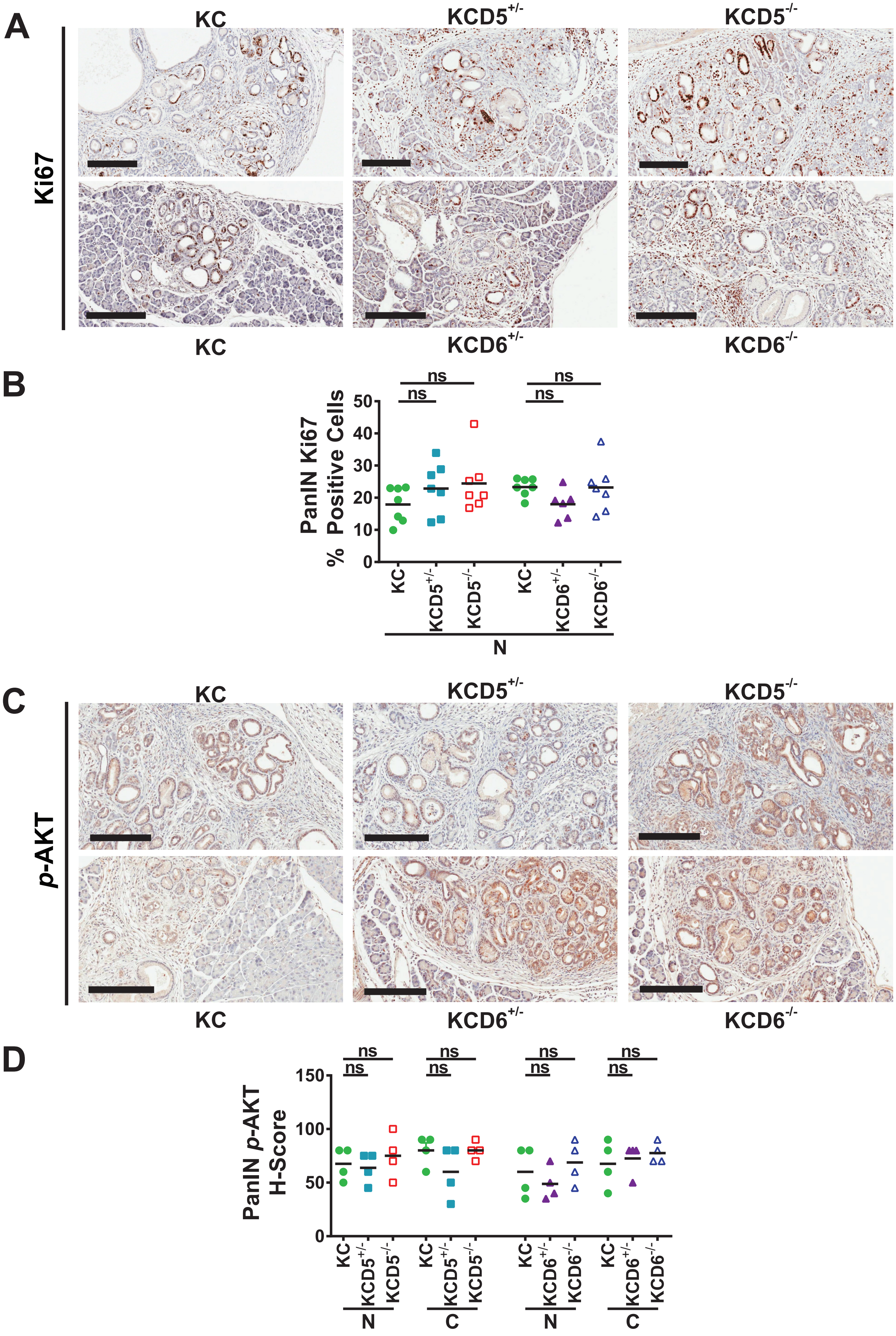
A
KC
KCD5+/-
KCD5-/-
Ki67
KC
KCD6+/-
KCD6-/-
B
ns
ns
ns
ns
PanIN Ki67
% Positive Cells
50
40
30
20
10
0
KC
KCD5+/-
KCD5-/-
KC
KCD6+/-
KCD6-/-
N
C
KC
KCD5+/-
KCD5-/-
p-AKT
KC
KCD6+/-
KCD6-/-
D
ns
ns
ns
ns
ns
ns
ns
ns
PanIN p-AKT
H-Score
150
100
50
0
KC
KCD5+/-
KCD5-/-
KC
KCD5+/-
KCD5-/-
KC
KCD6+/-
KCD6-/-
KC
KCD6+/-
KCD6-/-
N
C
N
C

Fig. S8

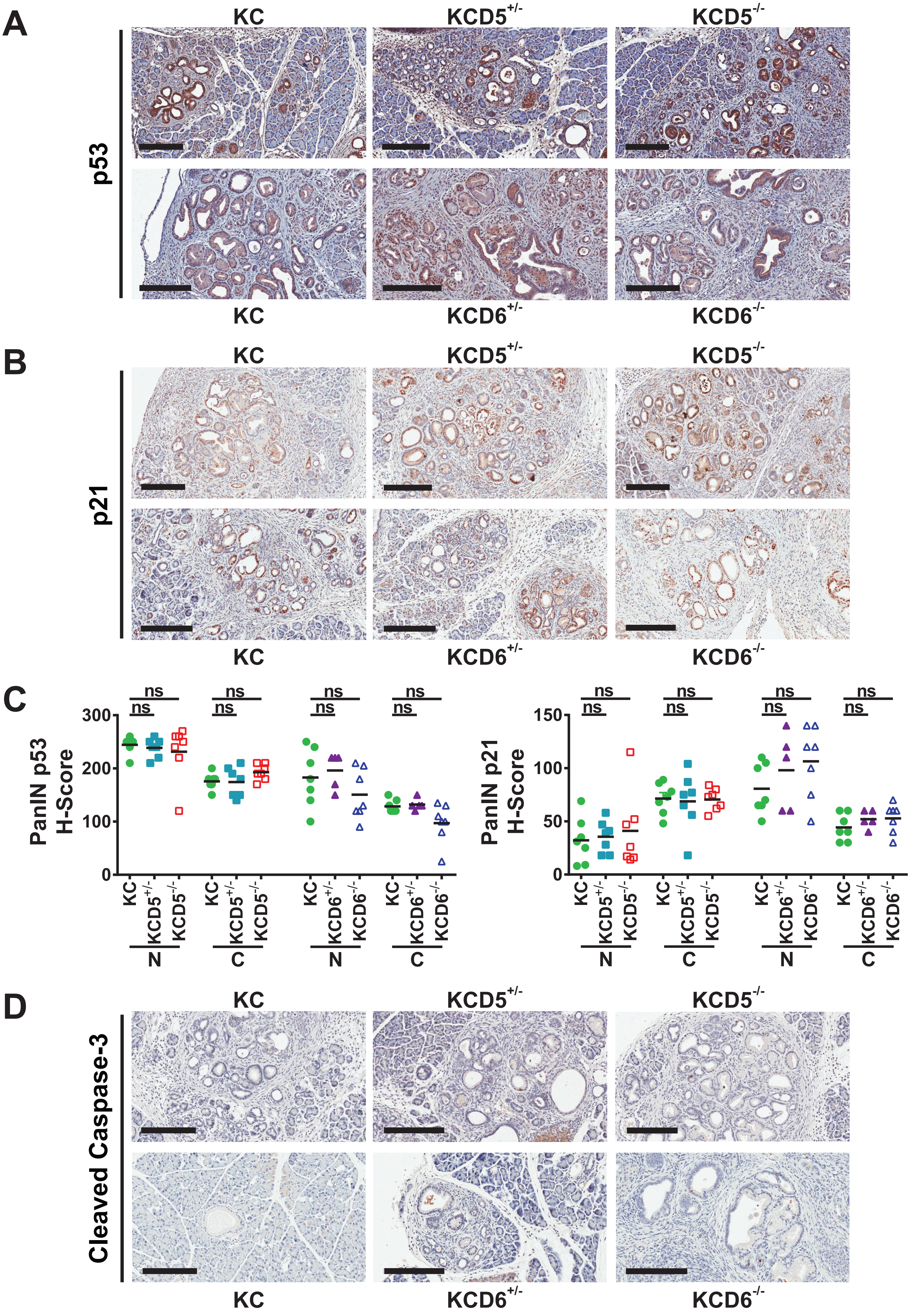
A
KC
KCD5+/-
KCD5-/-
p53
KC
KCD6+/-
KCD6-/-
B
KC
KCD5+/-
KCD5-/-
p21
KC
KCD6+/-
KCD6-/-
C
ns
ns
PanIN p53 H-Score
300
200
100
0
KC
KCD5+/-
KCD5-/-
KCD6+/-
KCD6-/-
N
C
PanIN p21 H-Score
150
100
50
0
D
KC
KCD5+/-
KCD5-/-
Cleaved Caspase-3
KC
KCD6+/-
KCD6-/-

Fig. S9

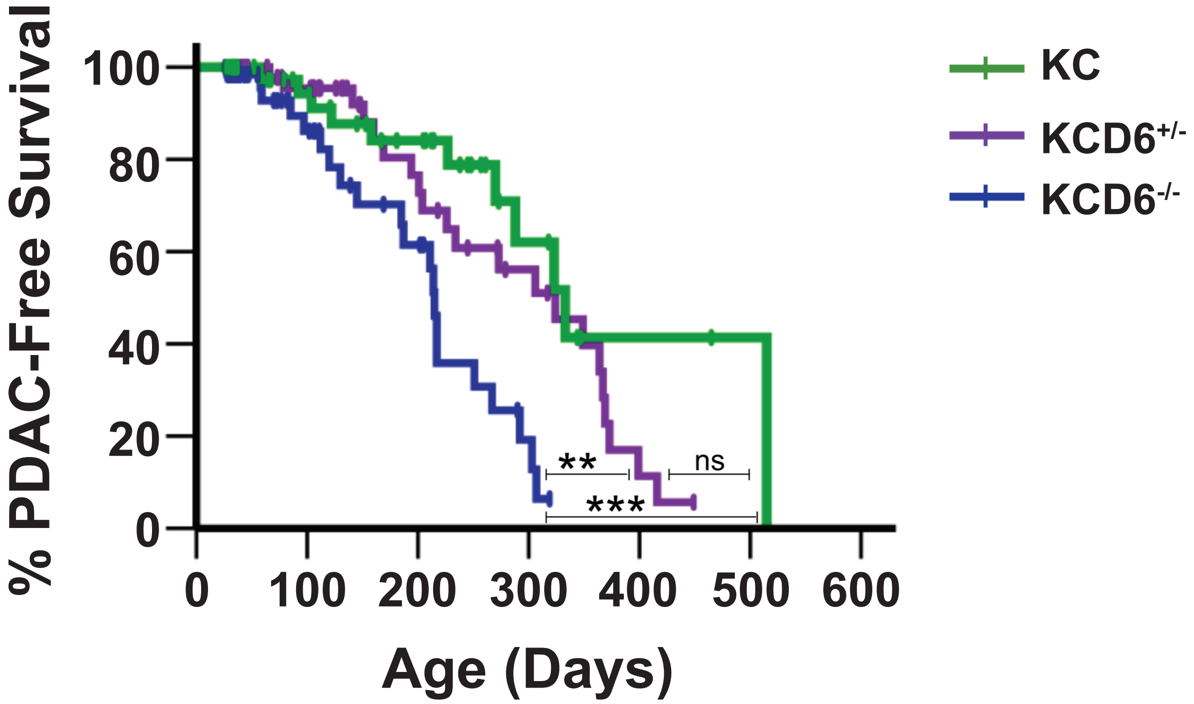
% PDAC-Free Survival
100
80
60
40
20
0
0
100
200
300
400
500
600
Age (Days)
KC
KCD6+/-
KCD6-/-
**
ns
***

Fig. S10

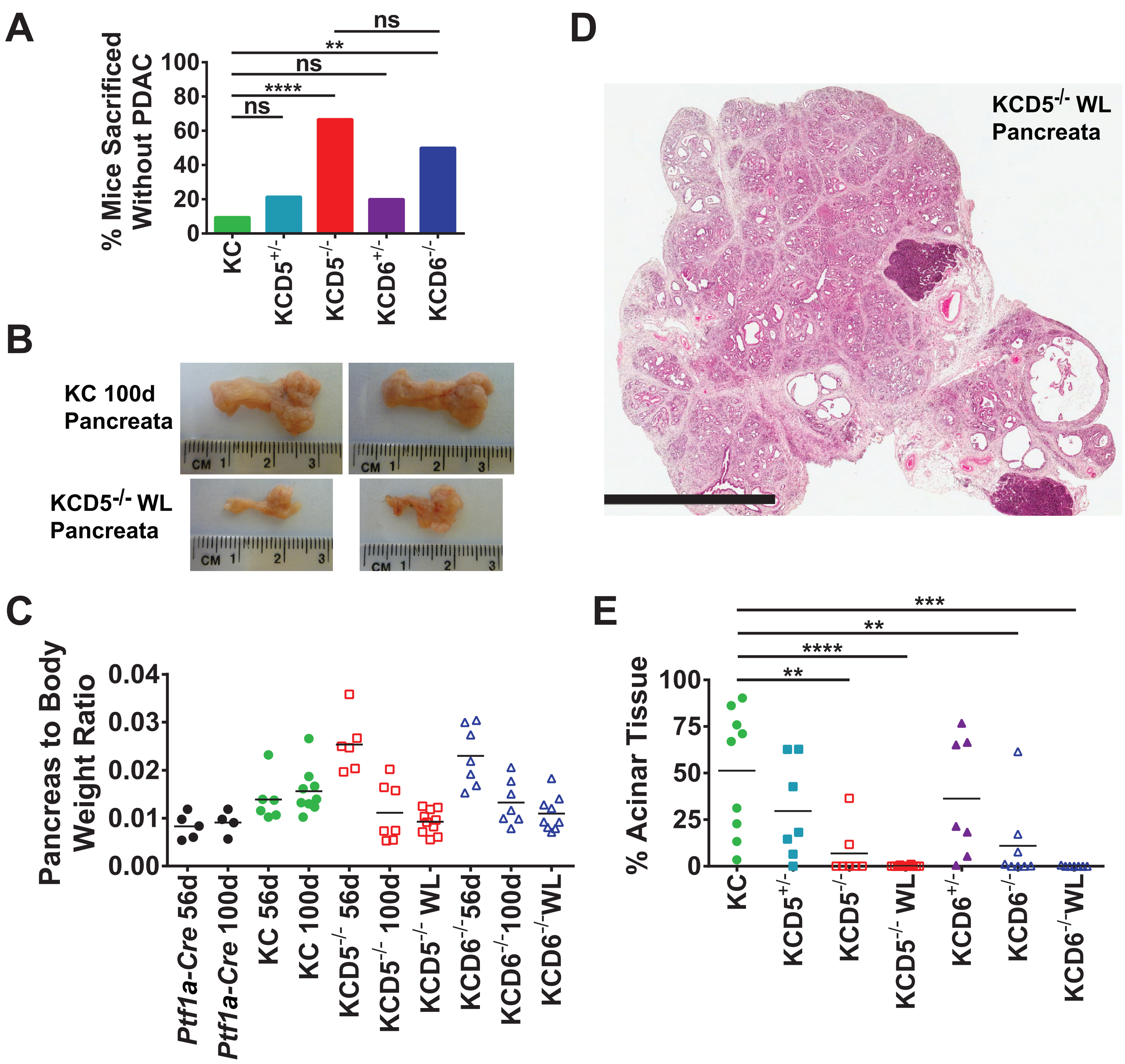
A
% Mice Sacrificed Without PDAC
100
80
60
40
20
0
ns
**
ns
****
ns
KC
KCD5+/-
KCD5-/-
KCD6+/-
KCD6-/-
B
KC 100d Pancreata
KCD5-/- WL Pancreata
CM 1 2 3
C
Pancreas to Body Weight Ratio
0.04
0.03
0.02
0.01
0.00
Ptf1a-Cre 56d
Ptf1a-Cre 100d
KC 56d
KC 100d
KCD5-/- 56d
KCD5-/- 100d
KCD5-/- WL
KCD6-/-56d
KCD6-/-100d
KCD6-/-WL
D
KCD5-/- WL Pancreata
E
% Acinar Tissue
100
75
50
25
0
***
**
****
**
KC
KCD5+/-
KCD5-/-
KCD5-/- WL
KCD6+/-
KCD6-/-
KCD6-/-WL

Fig. S11

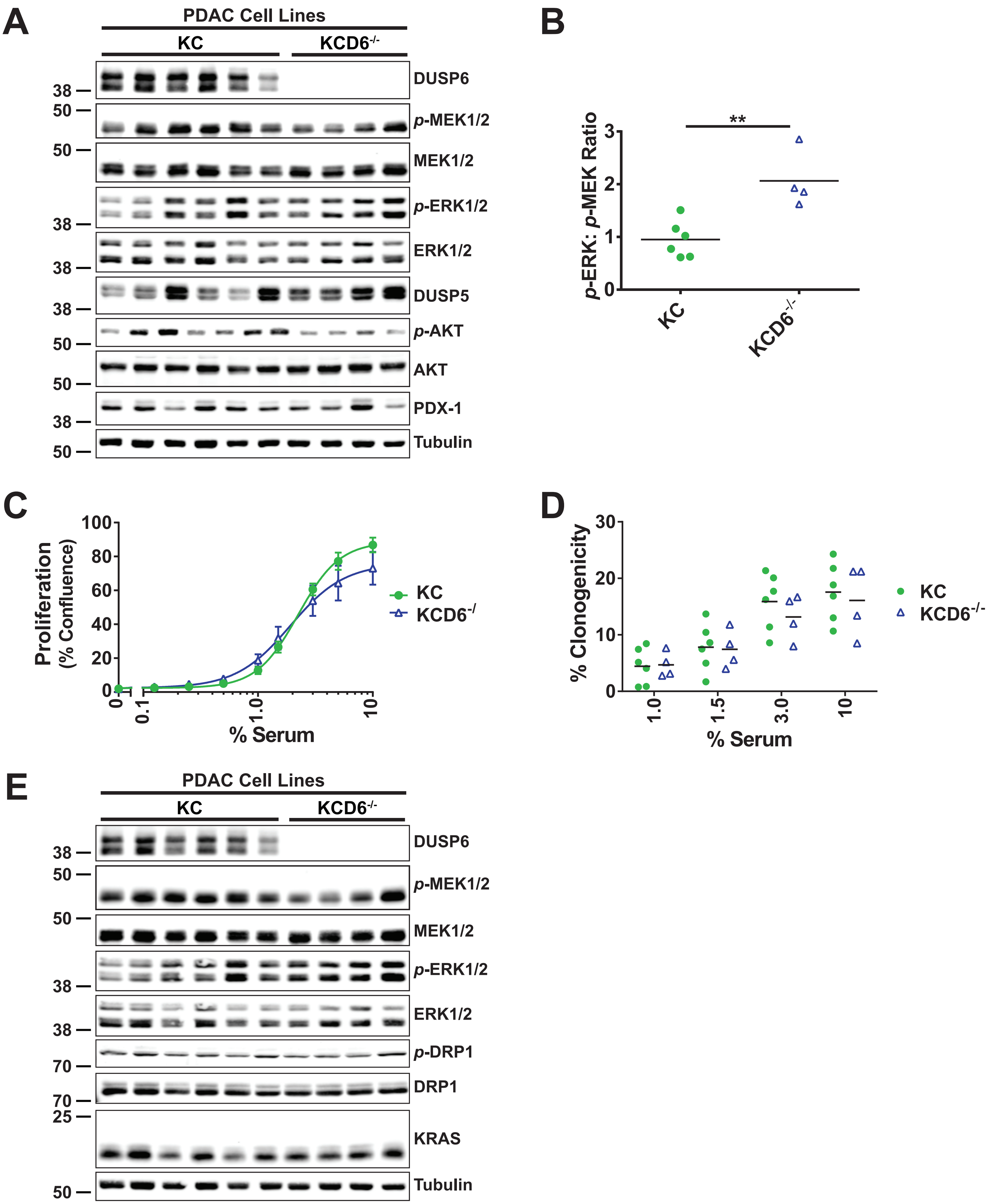
A
PDAC Cell Lines
KC
KCD6-/-
DUSP6
p-MEK1/2
MEK1/2
p-ERK1/2
ERK1/2
DUSP5
p-AKT
AKT
PDX-1
Tubulin
38
50
50
38
38
38
50
50
38
50
B
p-ERK: p-MEK Ratio
**
3
2
1
0
KC
KCD6-/-
C
Proliferation (% Confluence)
100
80
60
40
20
0
0
0.1
1.0
10
% Serum
KC
KCD6-/
D
% Clonogenicity
30
20
10
0
1.0
1.5
3.0
10
% Serum
KC
KCD6-/-
E
PDAC Cell Lines
KC
KCD6-/-
DUSP6
p-MEK1/2
MEK1/2
p-ERK1/2
ERK1/2
p-DRP1
DRP1
KRAS
Tubulin
38
50
50
38
38
70
70
25
50

Fig. S12

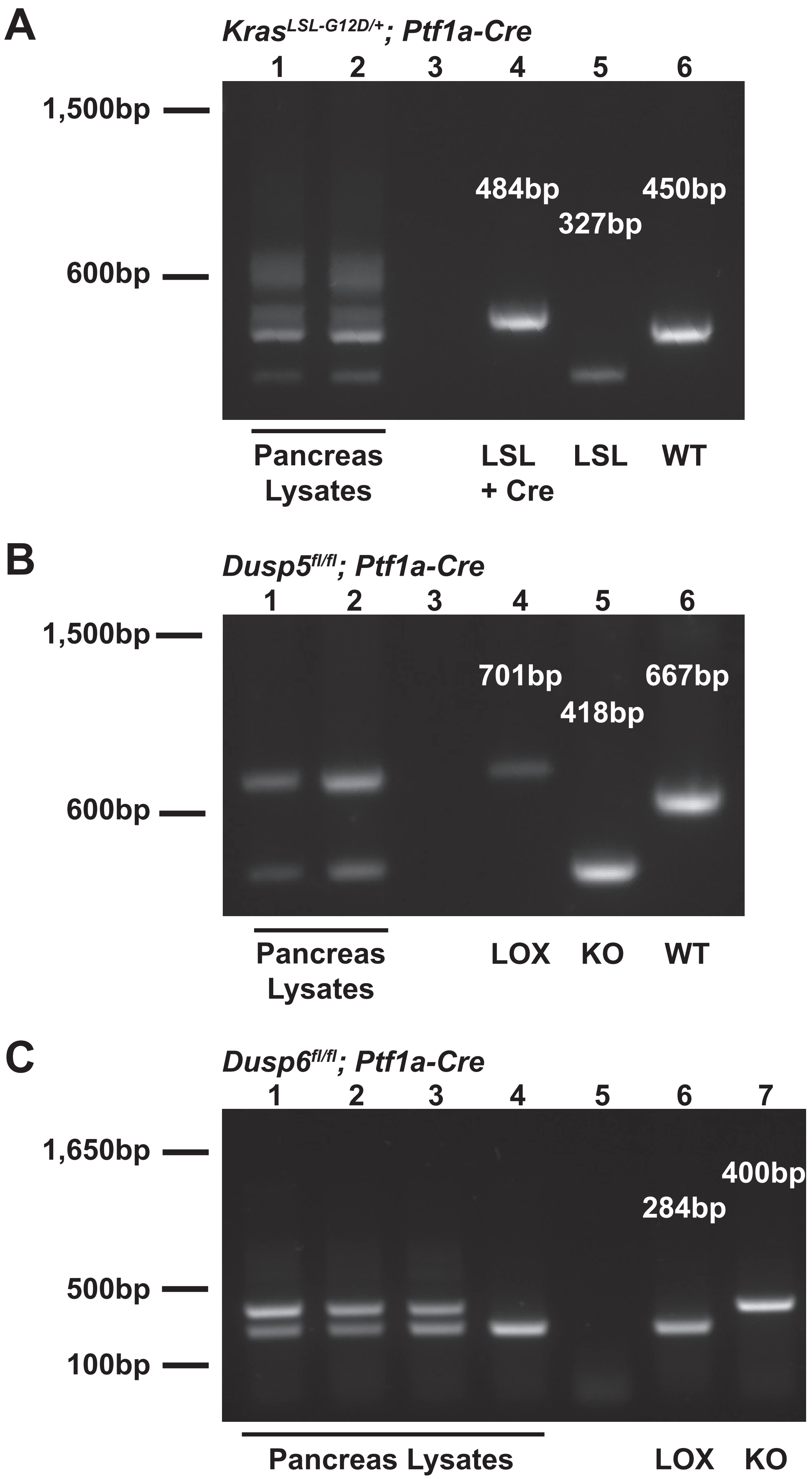
A
Kras^LSL-G12D/+; Ptf1a-Cre
1 2 3 4 5 6
1,500bp
600bp
484bp
327bp
450bp
Pancreas Lysates
LSL + Cre
LSL
WT
B
Dusp5^fl/fl; Ptf1a-Cre
1 2 3 4 5 6
1,500bp
600bp
701bp
418bp
667bp
Pancreas Lysates
LOX
KO
WT
C
Dusp6^fl/fl; Ptf1a-Cre
1 2 3 4 5 6 7
1,650bp
500bp
100bp
284bp
400bp
Pancreas Lysates
LOX
KO
